# Dual functions of TET1 in germ layer lineage bifurcation distinguished by genomic context and dependence on 5-methylcytosine oxidation

**DOI:** 10.1093/nar/gkad231

**Published:** 2023-04-06

**Authors:** Bernard K van der Veer, Lehua Chen, Colin Custers, Paraskevi Athanasouli, Mariana Schroiff, Riet Cornelis, Jonathan Sai-Hong Chui, Richard H Finnell, Frederic Lluis, Kian Peng Koh

**Affiliations:** KU Leuven, Department of Development and Regeneration, Laboratory of Stem Cell and Developmental Epigenetics, B-3000 Leuven, Belgium; KU Leuven, Department of Development and Regeneration, Laboratory of Stem Cell and Developmental Epigenetics, B-3000 Leuven, Belgium; KU Leuven, Department of Development and Regeneration, Laboratory of Stem Cell and Developmental Epigenetics, B-3000 Leuven, Belgium; KU Leuven, Department of Development and Regeneration, Laboratory of Stem Cell Signaling, B-3000 Leuven, Belgium; KU Leuven, Department of Development and Regeneration, Laboratory of Stem Cell and Developmental Epigenetics, B-3000 Leuven, Belgium; KU Leuven, Department of Development and Regeneration, Laboratory of Stem Cell and Developmental Epigenetics, B-3000 Leuven, Belgium; KU Leuven, Department of Development and Regeneration, Laboratory of Stem Cell Signaling, B-3000 Leuven, Belgium; Baylor College of Medicine, Department of Molecular and Cellular Biology, Center for Precision Environmental Health, Houston, TX 77030, USA; Baylor College of Medicine, Department of Molecular and Human Genetics, Department of Medicine, Houston, TX 77030, USA; KU Leuven, Department of Development and Regeneration, Laboratory of Stem Cell Signaling, B-3000 Leuven, Belgium; KU Leuven, Department of Development and Regeneration, Laboratory of Stem Cell and Developmental Epigenetics, B-3000 Leuven, Belgium; Baylor College of Medicine, Department of Molecular and Cellular Biology, Center for Precision Environmental Health, Houston, TX 77030, USA

## Abstract

Gastrulation begins when the epiblast forms the primitive streak or becomes definitive ectoderm. During this lineage bifurcation, the DNA dioxygenase TET1 has bipartite functions in transcriptional activation and repression, but the mechanisms remain unclear. By converting mouse embryonic stem cells (ESCs) into neuroprogenitors, we defined how *Tet1^–/–^* cells switch from neuroectoderm fate to form mesoderm and endoderm. We identified the Wnt repressor *Tcf7l1* as a TET1 target that suppresses Wnt/β-catenin and Nodal signalling. ESCs expressing catalytic dead TET1 retain neural potential but activate Nodal and subsequently Wnt/β-catenin pathways to generate also mesoderm and endoderm. At CpG-poor distal enhancers, TET1 maintains accessible chromatin at neuroectodermal loci independently of DNA demethylation. At CpG-rich promoters, DNA demethylation by TET1 affects the expression of bivalent genes. In ESCs, a non-catalytic TET1 cooperation with Polycomb represses primitive streak genes; post-lineage priming, the interaction becomes antagonistic at neuronal genes, when TET1’s catalytic activity is further involved by repressing Wnt signalling. The convergence of repressive DNA and histone methylation does not inhibit neural induction in *Tet1*-deficient cells, but some DNA hypermethylated loci persist at genes with brain-specific functions. Our results reveal versatile switching of non-catalytic and catalytic TET1 activities based on genomic context, lineage and developmental stage.

## INTRODUCTION

The study of mammalian cell fate acquisition in the early stages of embryonic development provides fundamental insights into the processes governing cellular plasticity. Recent single-cell atlases have comprehensively charted embryonic transcriptional changes to infer lineage segregation dynamics during mouse gastrulation at high resolution (1,2). In the gestational period between E6.5–7.5, the mouse epiblast differentiates dynamically and largely in a biphasic manner into either primitive streak fate, which subsequently gives rise to endoderm and mesoderm lineages, or else continue development to become definitive ectoderm. Whether this rapid lineage bifurcation process is regulated by stochastic and/or deterministic factors has been difficult to resolve. Classical models based on mouse development postulated the contribution of the extra-embryonic ectoderm as the origin of a bone morphogenetic protein (BMP)-Wingless/Integrated (Wnt)-Nodal growth signalling cascade that drives epiblast ingression into the primitive streak, suggesting that the developmental cues are cell-extrinsic (3). However, recent insights into the morphological events of primate and human gastrulation, together with *in vitro* differentiation models using embryonic stem cells (ESCs), suggest that germ layer lineage segregation can occur independently of extra-embryonic cells (4–6). A cell-intrinsic mechanism for controlling early cell fate choice may involve dynamic changes in the chromatin landscape – the epigenome comprising DNA methylation, histone modifications and non-coding RNAs – as the epiblast differentiates into a ‘formative’ pluripotent state, which confers developmental competence to respond to developmental signals at the onset of gastrulation (7,8). The extent by which DNA and histone methylation changes affect the earliest germ layer lineage bifurcation decision remains unclear (9,10).

Ten-Eleven-Translocation (TET) proteins are Fe^2+^ and α-ketoglutarate dependent dioxygenases that actively remove DNA methylation by reiterative oxidization of 5-methylcytosine (5mC) at CpGs to 5-hydroxymethylcytosine (5hmC) and further oxidized products (11–13). We previously demonstrated that TET1 is the dominant TET protein expressed between E5.5 to E6.5 in the pre-streak stage mouse embryo, when it regulates germ layer lineage bifurcation by promoting definitive ectoderm and repressing primitive streak fate entry (14–16). Others have also reported that loss of TET1 in mouse ESCs leads to both repression and activation of target genes (17–19). In mouse ESCs, TET1 and its product 5hmC are found associated not only with actively transcribed genes, but also with bivalent promoters marked by both active histone H3 lysine 4 trimethylation (H3K4me3) and repressive H3K27me3 chromatin states that keep most developmental lineage genes at low basal expression (18,20). Moreover, TET1 has been shown to co-occupy or interact with Sin3a/histone deacetylase (HDAC) and the Polycomb Group Repressive Complex 2 (PRC2) components Ezh2 and Suz12 (17–21). Recently, there has been more attention in dissecting the catalytic versus non-catalytic functions of TET1 in mouse ESCs. The interaction between TET1 and PRC2 has been validated to regulate embryonic development independently of TET1’s catalytic activity (22). Moreover, TET1 represses endogenous retroviruses independent of DNA demethylation (23). However, those studies have limited their analyses to naive ESC cultures. It remains unresolved how TET1’s dual function in transcriptional activation and repression is coordinated during lineage priming along bifurcating trajectories.

In this study, we used complementary bulk and single-cell sequencing methods to chart temporally changes in gene expression, chromatin states and DNA methylation caused by *Tet1* loss of function in an *in vitro* differentiation model that models neural induction. Our analysis unravelled a cell-intrinsic mechanism by which TET1 represses pre-mature primitive streak-inducing developmental signalling and dissected the nuanced contributions of TET1’s catalytic and non-catalytic activities in germ layer lineage bifurcation.

## MATERIALS AND METHODS

### ESC culture maintenance

All murine ESC lines were cultured on mitotically inactivated C57BL/6J (commonly known as B6) mouse embryonic fibroblasts (MEFs) in standard ESC culture medium composed of knock out DMEM (Invitrogen, 10829-018), 15% ESC-qualified fetal bovine serum (Invitrogen), 2 mM l-glutamine (Invitrogen, 25030-024), 0.1 mM 2-mercaptoethanol (Invitrogen, 31350-010), and 100:100 units:μg/ml penicillin:streptomycin (Sigma, P4333; or Gibco, 15140122), supplemented with leukaemia inhibitory factor (LIF) produced in-house as culture supernatant from a LIF over-expressing CHO-cell line. As feeder preparation, MEFs were cultured on 0.1% gelatin in MEF medium consisting of DMEM GlutaMAX (Invitrogen, 61965-026), 10% FBS (Sigma-Aldrich F7524), 2 mM l-glutamine, 1 mM sodium pyruvate, 0.1 mM each of nonessential amino acids, 0.1 mM 2-mercaptoethanol and 100:100 U:μg/ml penicillin:streptomycin. All lines were passaged using 0.25% trypsin–EDTA (Invitrogen, 25200072). When harvesting ESCs, feeders were depleted by 2 × 30 min re-plating on 0.1% gelatin-coated tissue culture plates.

For this study we used two littermate pairs of mouse blastocyst-derived *Tet1* knockout (KO) and wild-type (WT) (B6 × 129S6)F1-*Tet1*^tm1Koh^ male ESC lines (16), and one blastocyst-derived *Tcf7l1* KO line and R1 ESCs as a control with a similar genetic background (24). The genetic backgrounds of the (B6 × 129S6)F1 ESC lines have been characterized using a 384 SNP panel (Charles River lab), of which 196 SNPs are informative in discriminating B6 from 129S6 (also known as 129SvEvTac) allelic variants, as follows: WT7, 189 SNPs called, 60.6% B6/39.4%129S6; WT34, 189 SNPs called, 60.6% B6/39.4% 129S6; KO1, 190 SNPs called, 59.7% B6/40.3% 129S6; KO15, 190 SNPs called, 59.7% B6/40.3% 129S6.

### Creation of *Tet1* mutant cell lines

ESC WT7 was used as a parental line for generating a mutated version of *Tet1* (MUT) that is catalytically dead. We generated three *Tet1* MUT and three isogenic *Tet1* mock transfected clonal lines using Cas9 homology directed repair (HDR) as described in a previous study (25). Briefly, a single guide RNA (sgRNA) targeting the catalytic domain of TET1 in exon 11 was combined with Alt-R® S.p. HiFi Cas9 Nuclease V3 (IDT, 1081061) to form a Cas9 ribonucleoprotein (RNP) complex according to manufacturer's instructions. Cells were transfected with the assembled RNP complex (1.7 μM Cas9 nuclease, 2 μM sgRNA), 4 μM electroporation enhancer (IDT, 1075915) and 4.2 μM single stranded donor oligonucleotide (ssODN), using a mouse nucleofector kit (Lonza, VAPH-1001) on an Amaxa Nucleofector 2b (Lonza). Mock control cells were transfected without the RNP complex. After nucleofection, cells were seeded on feeders in standard ESC medium with 20 μM Alt-R® HDR Enhancer (IDT, 1081072). After 12h, medium was replaced with medium without HDR enhancer. Colonies were picked 72h after the transfection and screened for the edited using PCR amplification of the locus (345 bp), followed by restriction enzyme digestion with HaeIII (Bioké/NEB, R0108S). Clones with biallelic HDR edits were chosen for further analysis. For confirmation of HDR editing, gel-purified PCR products and 8 subcloned PCR fragments per clonal line were Sanger sequenced. All oligonucleotides used are listed in Supplementary Table S1.

### Creation of inducible-*Tcf7l1* ESC lines

For the creation of the doxycycline (DOX) inducible-*Tcf7l1* lines, WT7, KO1 and KO15 ESC lines were used as parental lines for lentiviral transduction. Lentiviruses were produced according to the RNAi Consortium (TRC) protocol available from the Broad Institute (https://portals.broadinstitute.org/gpp/public/resources/protocols). In brief, 7 × 10^5^ HEK293T cells were seeded per well in 6-well plates and transfected the following day with 0.75 μg pCMV-dR8.91, 0.25 μg pCMV-VSV-G, and 1 μg of the specific lentiviral expression constructs using FugeneHD (Promega, E2311) in Opti-MEM (Invitrogen, 31985070). One day after transfection, the culture medium was replaced with ESC medium supplemented with 0.01 ng/ml recombinant murine LIF (Peprotech, 250-02). The same day, ESCs were plated in 6-well plates on gelatin at a density of 10^5^ per well in the same medium. Lentivirus-containing medium was collected from HEK293T cells 48h and 72h after transfection and added in 1:1 ratio with fresh medium to recipient ESCs after being filtered. Two days after infection, ESCs were washed thoroughly with PBS, medium refreshed, and appropriate selection antibiotics applied for 10 days. First, parental lines were infected with pLVX-tet-On-Advanced construct and selected with 200 μg/ml G418 on neomycin resistant inactivated MEFs. After expansion of the lines, the ESCs containing the rtTA construct were infected with the pLVX-Tcf7l1 construct and selected with 2 μg/ml puromycin on SNLP 76/7-4 feeders. After selection, colonies were picked and screened for *Tcf7l1* induction after 48h of treatment with 2 μg/ml of DOX using RT-qPCR. Over expression of *Tcf7l1* after 48 h of 2 μg/ml DOX treatment in serum cultured ESCs on feeders was confirmed using western blots.

### Anterior neural progenitor cell (antNPC) differentiation

AntNPC differentiation was performed as previously described (16,26,27). Feeder depleted serum cultured ESCs were plated on gelatin coated plates at 10 000 cells/cm^2^ (90 000 cells per well in a 6-well plate) in advanced N2B27 defined media comprising a 1:1 mix of DMEM/F12 (Invitrogen, 12634-010) and Neurobasal medium (Invitrogen, 10888-022) supplemented with 0.5× B27 without vitamin A (Invitrogen, 12587–010), 0.5× N2 (Invitrogen, 17502-048), 2 mM l-glutamine (Invitrogen, 25030-024), 40 mg/ml BSA fraction V (Invitrogen, 15260037), 0.1 mM 2-mercaptoethanol (Invitrogen, 31350-010) and 100:100 units:μg/ml penicillin:streptomycin (Sigma-Aldrich, P4333; or Gibco, 15140122). Cells were supplemented with 10 ng/ml bFGF (Cat#100-18C, Peprotech) from day 0 until day 3. On day 3, the medium was changed to medium without bFGF. In some experiments, 5 μM of the Wnt inhibitor XAV939 (Sigma-Aldrich, X3004), 2.5 μM of the TGF-β receptor inhibitor SB431542 (Sigma-Aldrich, S4317), 0.1 μM BMP inhibitor LDN193189 (Sigma-Aldrich, SML0559), 0.3 μM or 3 μM GSK3 β inhibitor CHIR99021 (Axon Medchem BV,1408), 5 or 25 ng/ml of Activin A (Peprotechm 120-14E), or an equivalent amount of DMSO (vehicle control) was added from day 2 until day 5. The medium was changed every day during differentiation, except on day 1. At day 4, 1.5× amount of medium was added. The experimental end-point is day 5.

### Conversion of naive ESCs to formative epiblast-like cells (EpiLCs)

Conversion of ESCs toward EpiLCs was performed as described previously by Buecker et al. (2014), without supplementation of Activin A (15,16,28,29). EpiLCs are more recently characterized as a formative pluripotent state that models the E5–E6 pre-streak epiblast (7). Briefly, ESCs were first adapted for minimally five passages on to 2iL media conditions on gelatin. 2iL media consist of basal N2B27 medium as a 1:1 mixture of DMEM/F12 (Invitrogen, 11320-074) and Neurobasal medium (Invitrogen, 21103-049), 0.5× N2 (Invitrogen, 17502-048), 0.5× B27 (Invitrogen 17504-044), 2 mM l-glutamine, 0.1 mM 2-mercaptoethanol and 50:50 U:μg/ml penicillin/streptomycin, that is supplemented with 1 μM MEK inhibitor PD0325901, 3 μM GSK3 β inhibitor CHIR99021 and 0.01 ng/ml LIF (Peprotech, 250-02). To induce EpiLC differentiation, standard tissue culture plates were coated with 0.5 μg/cm^2^ fibronectin for 30 min and 3.0 × 10^4^ cells/cm^2^ were seeded in EpiLC conversion medium, which consists of basal N2B27 medium supplemented with 1% knockout serum replacement (KOSR) and 12 ng/ml bFGF. Medium was changed on day 1 and cells were harvested as EpiLCs on day 2.

### Dot blotting

Genomic DNA (gDNA) was extracted using PureGene genomic DNA extraction kit (Invitrogen, K182001) according to manufacturer's instructions. 200–250 ng of gDNA was serially diluted two-fold in nuclease-free water followed by denaturation in 0.4 M NaOH/10 mM EDTA at 95°C for 10 min, neutralized in equal volume of ice-cold 2 M ammonium acetate and kept on ice for 10 min. Samples were then spotted onto a Zeta-Probe nylon membrane (Bio-Rad, Cat #162-0165) with a 96-well Bio-Rad Bio-Dot apparatus. The spotted membrane was subsequently washed excessively with 0.4 M NaOH, air-dried for 5–10 min and UV cross-linked two times at 120 000 μJ/cm^2^ on a UVP HL-2000 HybriLinker. The membrane was then blocked in 5% non-fat dry-milk in Tris-buffered saline with 0.1% Tween-20 (TBS-T) for 1 h and incubated overnight at 4°C with primary antibodies diluted in 5% non-fat dry-milk in TBS-T. Antibodies used were: anti-5hmC (Active Motif, 39769; 1:10 000) and anti-dsDNA (Abcam, ab27156, 1:1000; which cross-reacts with both single and double-stranded DNA). Subsequently, membranes were incubated with secondary antibodies diluted 1:5000 in 5% non-fat dry milk in TBS-T (HRP-conjugated anti-rabbit, Dako, P0217, 1:5000; or HRP-conjugated anti-mouse, Dako, P0447, 1:5000). The signal was detected using enhanced chemiluminescence using ClarityWestern ECL substrate (Bio-Rad 1705060) captured using light-sensitive film and developed using an AGFACurix 60 Film Processor (Bio-Rad). On every membrane, a serial dilution of 0.1 ng DNA of 5hmC PCR product was spotted. To assess the quantities of 5hmC, signal intensities of 5hmC were measured using FIJI (ImageJ 1.53c) software and calibrated against the linear range of the standard curves.

### Western blot

Cell pellets were lysed in RIPA buffer (50 mM Tris at pH 8.0, 150 mM NaCl, 0.2 mM EDTA, 1% NP-40, 0.5% sodium deoxycholate, 0.1% SDS) containing 1 mM phenylmethylsulfonyl fluoride, 0.5 mM DTT, phosphatase inhibitor cocktail 2 and 3 (Sigma-Aldrich, P5726 and P0044) and protease inhibitor cocktail (Roche, 11836153001) on ice. Lysates were passed through a 26-gauge (26-G) needle and centrifuged 15 min at 16 000 x *g* at 4°C and supernatant was collected and stored at –80°C. Protein concentration was measured using Bradford assay in a 96-well micro plate format. Protein samples were prepared in 1× Laemmli sample buffer (62.5 mM Tris–HCl at pH 6.8, 2.5% SDS, 0.002% bromophenol blue, 5% β-mercaptoethanol, 10% glycerol) by boiling for 10 min at 95°C. 10–20 μg of protein was loaded on an 8% (for TET proteins) or 10% (other proteins) SDS–polyacrylamide gel. Samples were run in 1× running buffer (25 mM Tris, 192 mM glycine, 0.1% SDS) and then transferred to a PVDF membrane with transfer buffer (25 mM Tris, 192 mM glycine and 20% methanol; but 10% methanol plus 0.1% SDS when transferring TET proteins). Membranes were blocked with 5% non-fat dry milk in TBS-T, or in 5% BSA in TBS-T specifically for phospho-proteins and TCF7L1. Subsequently, primary antibodies were diluted in 5% milk or 5% BSA (depending on blocking) and incubated overnight at 4°C, followed by incubation with corresponding secondary antibodies conjugated to HRP (diluted 1:5000 in TBS-T with 5% nonfat milk) for 1 h at room temperature. The signal was detected using ClarityWestern ECL substrate (Bio-Rad 1705060) on AGFACurix 60 Film Processor. Signal intensity of was measured using FIJI (ImageJ v1.53c). Primary antibodies used in this study are: anti-TET1 (Genetex, GTX125888, 1:1000), anti-TET2 (Abcam, ab94580, 1:1000), anti-TET3 (Santa-Cruz, sc-139186, 1:1000), anti-TCF7L1 (Santa-Cruz, sc-166411) anti-non-phospho(Ser33/37/Thr41) (active)-CTNNB1 (Cell Signaling Technology, 8814, 1:1000), anti-(total) CTNNB1 (Biosciences, 610153, 1:1000), anti-phospho(Ser465/467)-SMAD2 (Millipore, ab3849-I, 1:1000), anti-(total) SMAD2/3 (Cell Signaling Technology, 3102 1:1000), anti-ACTB (Sigma-Aldrich, A1978, 1:2000), anti-αTUB (Cell Signaling Technology, 2144, 1:1000).

### Immunofluorescence

Cells were cultured on sterilized coverslips or regular tissue culture plates. To stain, cells were fixed in 4% PFA solution for 10 min at room temperature and permeabilized with 0.5% Triton X-100 in PBS, followed by two washes with 0.2% Tween in PBS (PBS-T). Blocking was performed 1 h at room temperature with blocking solution (10% normal donkey serum, 0.2% Tween-20, 2% fish gelatin in PBS) and followed by overnight incubation in a moist chamber at 4°C with primary antibodies (anti-T, Santa Cruz Biotechnology sc17743, 1:200; anti-MEF2C, Abcam, ab211493, 1:500) diluted in antibody solution (1% normal donkey serum, 0.2% Tween-10, 0.2% fish gelatin in PBS. Corresponding secondary antibodies (1:500) diluted in antibody solution were incubated for 1 h at room temperature, followed by a wash with PBS-T, counterstaining of the nuclei with 0.1 μg/ml DAPI in PBS-T for 5 min, followed by a wash with PBS-T and PBS. Coverslips were mounted with Prolong Gold and imaged using a Nikon Eclipse Ti-2 microscope. Images were processed using FIJI (ImageJ v1.53c).

### TOP-flash dual-luciferase assay

On day 1 of differentiation, cells were transiently transfected with a mix of Opti-MEM I Reduced-Serum Medium (Thermo Fisher 31985047), 2.5 μg of plasmid DNA and 6 μl of TransIT-LT1 Transfection Reagent per reaction in a standard 6-well plate. The DNA was a mix of 2.25 μg TOP/FOP-Flash reporter and 0.25 μg pRL-TK (30). The Dual-Luciferase Reporter Assay system (Promega, E1910) was used for collection and luminescence measurement, according to manufacturer's instructions. In short, on day 3 or 4 of differentiation the cells were washed with PBS and 500 μl Passive Lysis Buffer (provided in the kit) was added and incubated for 15 min on an orbital shaker at room temperature. Cells were scraped and lysates were passed through a 26G needle to aid in lysis and homogenization. 20 μl of each lysate was used to measure both firefly luciferase and *Renilla* luciferase activity in a white 96-well plate (Perkin Elmer, 6005500) using the Biotek Synergy HTX multi-mode reader with a 10 s measurement period for each sample per assay (firefly or *Renilla*). Every sample was measured in technical triplicates.

### Quantitative reverse transcription-polymerase chain reaction (RT-qPCR)

Total RNA was extracted using Trizol (Invitrogen, 15596018) or RNeasy plus mini kit (Qiagen, 74136) according to the manufacturer's instructions. 200 ng–1 μg of RNA was used for reverse transcription reactions using the Superscript III cDNA synthesis (Thermo Fisher 11752-050) followed by RNase treatment, according to the manufacturer's instructions. Quantitative real time PCR reactions were set up in technical triplicates using cDNA diluted 1:2–1:10 (depending on RNA input) and SYBR-green PCR master mix (Thermo Fisher 11733-046) and 5 nM primers (Supplementary Table S1) using a 384 wells ViiA7 real-time PCR system (Applied Biosystems). Expression levels of target genes was calculated according to the 2^–ΔΔCt^ method using QuantStudio Real-Time PCR software (v1.3). *Gapdh* was used as a reference gene for normalization and fold induction was calculated relative to expression in control ESCs.

### Chromatin immunoprecipitation (ChIP) qPCR

5 × 10^6^ cells per ChIP-qPCR was chemically cross-linked with 1% methanol-free formaldehyde (Polysciences, 04018) for 10 min at RT and then quenched with 0.125 M glycine for 10 min at RT. Fixed cells were lysed sequentially in Buffer I (50 mM HEPES–KOH at pH 7.5, 140 mM NaCl, 0.25% Triton X-100, 0.5% NP-40, 10% glycerol, 1 mM EDTA), Buffer II (10 mM Tris–HCl at pH 8.0, 200 mM NaCl, 1 mM EDTA, 0.5 mM EGTA) and Buffer III (10 mM Tris–HCl at pH 8.0, 100 mM NaCl, 1 mM EDTA, 0.5 mM EGTA, 0.1% sodium deoxycholate, 0.5% *N* -lauroylsarcosine). Chromatin was sheared to 200–500 bp using Q800R3 Sonicator under a high-power setting for 20 cycles (30 s on, 30 s off) at 4°C. The sheared DNA was incubated with 2.5 μg of antibody per ChIP (TET1, GeneTex GTX124207; SUZ12, Cell Signaling Technologies 3737S), rotating overnight at 4°C and then precipitated with Protein G Dynabeads (Thermo Scientific 10004D). Precipitates were washed sequentially using the following solutions for 5 min each: low-salt buffer (20 mM Tris–HCl at pH 8.0, 150 mM NaCl, 2 mM EDTA, 1% Triton X-100, 0.1% SDS), high-salt buffer (20 mM Tris–HCl at pH 8.0, 500 mM NaCl, 2 mM EDTA, 1% Triton X-100, 0.1% SDS), LiCl buffer (10 mM Tris–HCl at pH 8.0, 250 mM LiCl, 1 mM EDTA, 1% deoxycholate, 1% NP-40), and twice in TE buffer + 50 mM NaCl. Chromatin antibody beads were eluted in 50 mM Tris–HCl (pH 8.0), 10 mM EDTA, and 1% SDS at 65°C for 2 hours and de-cross-linked in 5 M NaCl solution at 65°C overnight. Chromatin extracts were incubated with DNase-free RNase (Roche, 04716728001) for 30 min at 37°C and afterward with 10 mg/ml proteinase K (Roche, 03115879001) for 2 h at 45°C. Sheared DNA was purified using the QIAquick PCR purification kit (Qiagen, 28106) for qPCR preparation. Quantitative PCR reactions were set up in technical triplicates using chromatin extracts diluted 1:10 and SYBR-green PCR master mix (Thermo Fisher 11733-046) and 5 nM primers (Supplementary Table S1) using a 384 wells ViiA7 real-time PCR system (Applied Biosystems). We used standard curves generated with known amounts of serially diluted genomic DNA for quantification of TET1 occupancy using QuantStudio Real-Time PCR software (v1.3), normalized to input per sample. *Npr3* was used as the negative control locus in TET1 and SUZ12 ChIP-qPCR.

### Embryo isolation

*Tet1* ^tm1Koh^ heterozygous males and females were naturally mated to generate embryos (15). E8.5 and E11.5 embryos were collected from timed-pregnant females. The morning a copulation plug was found, is considered as E0.5. Individual decidua were collected in a dish with cold PBS, and uterine tissue was removed with a fine pair of dissection scissors, followed by extraction of embryos using a of sharp Dumont #5 forceps. Embryonic brain or anterior neural tissues were dissected as previously described (15,31). Dissected tissues were snap frozen in liquid nitrogen and stored at –80°C for later processing. For E8.5 samples, the remainder of the embryo was used for genotyping, while for E11.5, the yolk sac was used. Primers for both genotyping of *Tet1* and sex are listed in Supplementary Table S1.

### Targeted amplicon bisulfite seq

Genomic DNA (gDNA) was extracted from minimally 10^6^ cells or E11.5 brains using the Purelink genomic DNA Mini Kit (Invitrogen, K182001), according to the manufacturer's instructions. The quality of the gDNA was assessed using Nanodrop and the absence of RNA contamination was checked by running the samples on a 0.8% agarose gel stained with SyberSafe. gDNA was extracted from E8.5 anterior neural (headfold) tissues (roughly 10^4^ cells) by incubating the tissues with lysis buffer (10 mM Tris–HCl pH 8.3, 50 mM KCl, 2.5 mM MgCl_2_, 0.5% NP-40) and 0.4 mg/ml Proteinase K (Thermo Fisher Scientific, AM2546) for 1 h at 56°C and subsequently 30 min with 2 mg/ml RNAse A (Qiagen, 19101) at 37°C. After a 1× clean-up using AMPure XP beads (Beckman Coulter, A63881), gDNA was eluted in 20 μl (100–200 ng yield) and used entirely for bisulfite conversion. For other samples, 1.5 μg of gDNA was used for bisulfite conversion, using the EpiTect Fast DNA Bisulfite kit (Qiagen, 59824) and eluted in 15 μl of elution buffer provided in the kit, according to the manufacturer's specifications. A separate 20 μl PCR reaction was used for each amplicon with 0.5 μl of bisulfite converted gDNA, 300 nM of both a forward and reverse primer containing P7 and P5 tails, and Platinum™ *Taq* DNA polymerase High Fidelity (Invitrogen, 11304-011) and provided buffers. PCR cycling parameters are listed in Supplementary Table S1. Amplicons were loaded on a 1.5% agarose gel and individual bands were gel-extracted using PureLink Quick Gel Extraction kit (Invitrogen, K210012). The concentration of each amplicon was measured using Qubit™ dsDNA HS Assay kit (Invitrogen, Q32854) and diluted to 15 nM. The amplicons were pooled equimolar per sample and used for secondary PCR reaction to generate libraries.

The quality of the pooled amplicons was assessed using fragment analyzer (Agilent) and the Qubit™ dsDNA HS Assay kit (Invitrogen, Q32854). The amplicon pools were diluted to max 5 ng/μl and combined as follows: 9 μl DNA, 0.5 μl custom p7 primer (125 nM), 0.5 μl custom p5 primer (125 nM) and 1 × 10 μl Phusion® High Fidelity PCR master Mix with HF buffer (Biolabs new England M0531S). The following program was used in a thermocycler: 94 °C 30 sec; 15 × 94 °C 10 s, 51 °C 30 s, 72 °C 30 s; 72 °C 1 min. The custom primers are provided with unique dual indexes to label the samples. The resulting library was purified with a 1× clean-up using AMPure XP beads following manufacturers protocol. The final quality of libraries was analysed using fragment analyzer (Agilent) and pooled equimolar. The concentration of the final pool was measured using qPCR (Kapa sybr fast, Roche, KK4600) and loaded on a NovaSeq for PE150 sequencing for a minimum of 200 000 reads per amplicon and on average 350 000.

Using Trim Galore! (v0.6.7), reads were trimmed based on quality (PHRED < 20), adapters were removed and only reads with a minimum length of 20 bp were kept. Using Bismark (v0.23.1), the trimmed reads were aligned to GENCODE mm10 (GRCm38.p6) with a maximal insert size of 500 bp, followed by methylation extraction. Only CpGs were kept with a minimal coverage of 1000× and plotted over the seven different assayed regions using a custom script in R (v4.0.3).

### RNA-seq library preparation

Total RNA was extracted from cells using Trizol using the manufacturer's instructions. RNA sequencing (RNA-seq) libraries were prepared from 4 μg of total RNA using the KAPA stranded mRNA-seq kit (Roche, KK8421) according to manufacturer's specifications. 100 nM KAPA-single index adapters (Roche, KK8700) were added to A-tailed cDNA, and libraries were amplified for 10 cycles. Finally, 1x library clean-up was performed using Agencourt AMPure XP beads (Beckman Coulter, A63881). Library fragment size was assessed using Agilent Bioanalyzer 2100 with the High Sensitivity DNA analysis kit (Agilent, 5067-4626) and concentration was determined using Qubit™ dsDNA HS Assay kit (Invitrogen, Q32854). Each library was diluted to 4 nM and pooled for sequencing on an Illumina Hiseq4000, aiming at 15–20 million SE50 reads per sample (19 million reads on average).

### 10xGenomics Single-cell (sc)RNA-seq library preparation

For single-cell RNA-seq library preparations, cells were washed with PBS and incubated for 5 min with TrypLE, which was subsequently quenched with advanced DMEM/F12. Single cells were washed 3x with 0.4% BSA in PBS. Cells were counted using the LUNA counter and an AO/PI fluorescent stain. Cells were loaded to target 5000 following the 10xGenomics protocol for single cell 3’ prime reagent kit v3.1 (single indexes). Quality control was performed and pooled according to instructions and sequenced following the 10xGenomics guidelines at 28–8–0–91 on a NovaSeq 6000 Instrument. First a shallow sequencing run was performed to perform quality control on the libraries and estimate the number of captured cells, followed by a deeper sequencing run to reach a mean of 20 000 reads per cell. In total we captured 54 708 high quality cells after filtering, with on average 5471 cells per sample.

### oxWGBS library preparation

Genomic DNA was extracted from minimally 10^6^ cells using the Purelink genomic DNA Mini Kit (Invitrogen, K182001), according to the manufacturer's instructions. The quality of the gDNA was assessed using Nanodrop and absence of RNA contamination was checked by running the samples on a 0.8% agarose gel stained with SYBER Safe. Per sample, 1.5 μg of gDNA in a total volume of 50 μl was sheared using COVARIS M220 for 2 × 60 s with at an intensity of 5 at 7°C followed by a 1.8× clean-up using AMPure XP beads (Beckman Coulter, A63881) to concentrate fragmented DNA. Fragmentation was assessed Agilent Bioanalyzer 2100 with the High Sensitivity DNA analysis kit (Agilent, 5067-4626) and concentration was determined using Qubit™ dsDNA HS Assay kit (Invitrogen, Q32854).

Libraries were made and (oxidative) bisulfite converted using the Ultralow Methyl-Seq kit (Tecan/Nugen), according to the manufacturer's instructions. For BS and oxBS, libraries were made in parallel starting from the same fragmented gDNA per sample. For each library, 300 ng of fragmented gDNA was used as input and fragments were end repaired and ligated to single-indexed sequencing adapters provided in the kit, followed by final repair. Before oxidation and bisulfite conversion, libraries were purified and washed 3× with 80% acetonitrile to get rid of any residual ethanol, followed by incubation at 37°C for 5 min with denaturing buffer provided with the kit to denature the DNA. oxBS libraries were oxidized by addition of TrueMethyl oxidation solution while BS libraries were mock treated using ultra-pure water during an incubation at 40°C for 10 min. For each library, the optimal amplification was optimized using qPCR; 1/6th of the libraries was added to amplification master mix containing SYBR Green and run on an Applied Biosystems StepOnePlus Real-Time PCR system for 30 cycles. Relative log-fluorescence vs amplification cycle was plotted out to manually determine the appropriate amplification cycles, selected within the middle to late exponential phase of amplification. The BS libraries were amplified between 7 and 12 cycles, while oxBS libraries were amplified between 10 and 15 cycles. Following amplification, bisulfite converted libraries were purified finally with a 1× clean-up using AMPure XP beads. The quality of the libraries was assessed using an Agilent Bioanalyzer 2100 with the High Sensitivity DNA analysis kit (Agilent, 5067-4626) and concentration was determined using Qubit™ dsDNA HS Assay kit (Invitrogen, Q32854). The libraries were first sequenced shallow to estimate library quality, bisulfite conversion efficiency, and duplicate rate, followed by multiple deep sequencing PE150 runs to obtain an average coverage of 5× per library. For sequencing the custom sequencing primer MetSeq Primer 1 was used, as per manufacturer specification. Bisulfite conversion rate was >99% for each library.

### ATAC-seq library preparation

We used the Omni-ATAC protocol to prepare ATAC-seq libraries as previously described (32,33). Although viability of the cells was high (>90–95%), we treated them nonetheless by adding 0.1 mg/ml DNase I (Worthington, LS002005) directly to the growth medium supplemented with 0.5 mM CaCl_2_, 2.5 mM MgCl_2_, and incubating for 30 min at 37 °C. Cells were then washed with PBS and incubated for 5 min with TrypLE which was subsequently quenched with medium. After two washes with ice-cold PBS, 50 000 cells were pelleted in a pre-cooled 1.5 ml tube by centrifugation for 5 min at 600 x *g* at 4 °C in a microcentrifuge. Supernatant was removed and 50 μl of ATAC-lysis buffer was added (10 mM Tris–HCl pH 7.4, 10 mM NaCl, 3 mM MgCl_2_, 0.1% NP40, 0.1% Tween-20, 0.01% digitonin) and incubated on ice for 3 min, followed by addition of 1 ml of ATAC-wash buffer (ATAC-lysis buffer, without NP40 and digitonin) and the tube was inverted 3 times. The nuclei were pelleted for 10 min at 600 rcf at 4°C in a microcentrifuge. Supernatant was removed, and nuclei were resuspended in 50 μl transposition reaction buffer (5 μl H_2_O, 15 μl PBS, 0.5 μl 10% Tween-20, 0.5 μl 1% digitonin, 25 μl TD buffer, 2.5 μl Tagment DNA Enzyme 1) (Tagment DNA Enzyme and Buffer Small Kit, Illumina, 20034197) and incubated for 30 min at 37 °C at 1000 rpm in a thermomixer. The transposed DNA was cleaned up immediately following transposition using the ZymoDNAClean & Concentrator-5 kit (Zymo D4014) following manufacturer instructions.

The transposed DNA was amplified in one pre-amplification step (5 cycles) and a final amplification (5–7 cycles) in the same 50 μl reaction using 1× NEBNext High-fidelity PCR master mix (Bioké/NEB, M0541S) and 125 nM of Ad1_noMX primer and Ad2.index primer with the following program: 72°C 5 min, 98°C 30 s; 5 to 7 × 98 °C 10 s, 63 °C 30 s, 72 °C 1 min; 72 °C 1 min. For each library, the optimal amplification was optimized using qPCR; 1/5th of the pre-amplified libraries was added to amplification master mix containing SYBR Green and run on an Applied Biosystems StepOnePlus Real-Time PCR system for an additional 20 cycles. Relative log-fluorescence versus amplification cycle was plotted out to manually determine the appropriate amplification cycles. The number of cycles was selected to reach 1/3 of the final relative fluorescence unit value.

Following amplification, the libraries were purified using ZymoDNAClean& Concentrator-5 kit (Zymo D4014) followed by 0.55×–1.75× dual size selection using AMPure XP beads. The quality of the libraries was assessed using an Agilent Bioanalyzer 2100 with the High Sensitivity DNA analysis kit (Agilent, 5067-4626) and concentration was determined using Qubit™ dsDNA HS Assay kit (Invitrogen, Q32854). The libraries were diluted to 4 nM and pooled together for SE50 or PE50 sequencing on an Illumina Hiseq4000, aiming for minimally 25 million mapped and unique reads.

### Cleavage Under Target & Release Using Nuclease (CUT&RUN) library preparation

CUT&RUN was performed using the CUTANA™ KIT (Epicypher, 14-1048) according to the manufacturer's instructions with minor modifications (34,35). Briefly, 5 × 10^5^ cells were collected as a single cell suspension and washed three times with 100 μl/sample wash buffer (20 mM HEPES pH 7.5, 150 mM NaCl, 0.5 mM Spermidine and 1 Roche complete tablet/50ml). Cells were then bound to activated Concanavalin A beads and incubated for 10 min at RT, followed by incubation with 0.5 μg anti-H3K27me3 (Invitrogen, MA5-11198) antibody or IgG (Epicypher, 18-1401) diluted in antibody buffer on a nutator overnight at 4°C. After three washes of magnet-bound beads with digitonin buffer, pAG/MNase was added and incubated for 10 min at RT, washed and resuspended in digestion buffer for a 30-minute incubation on ice. The reaction was stopped by addition of STOP buffer and incubated for 10 min at 37°C. The supernatant containing enriched DNA was then extracted using the supplied DNA Clean-up Columns. Libraries were prepared using the NEBNext® Ultra II DNA library Prep Kit for Illumina (New England Biolabs®, NEB E7645L) according to Epicypher's instructions, and amplified by PCR using NEBNext Dual Index Primers (New England Biolabs, E6440S) with the following settings: 98°C 45 s; 14 × 98°C 15 s, 60°C 10 s; 72°C 1 min. Library concentration was quantified using Qubit HS dsDNA Quant kit (ThermoFisher, Q32851). Quality and size distribution were further assessed on an Agilent 2100 Bioanalyzer using a High Sensitivity DNA Analysis Kit (Agilent, 5067-4626). Libraries were diluted to 4 nM and pooled together for PE50 sequencing on NextSeq500 or NextSeq2000 for minimally 5 million reads per sample.

### RNA-seq analysis

Adapters, polyA/T tails, and bad quality reads (Phred score > 20) were trimmed using Trim Galore! (v0.6.4_dev) with default parameters. Reads were aligned to the transcriptome and quantified using Salmon (v0.14.1) (36) with default parameters using GENCODE release 23 of the mouse reference transcriptome sequences and the comprehensive gene annotation. Subsequently, the counts were imported into R (v4.0.2) using tximport (v1.18.0). Differentially expressed genes were defined using DEseq2 (v1.30.0) (37) and log fold changes corrected using ‘ashr’ method (FDR adjusted *P*-value <0.05 and |log_2_(fold change)| > 1.5) (38). Temporal differentially expressed genes were determined using ImpulseDE2 (v1.10.0) with default settings. GO term enrichment was performed using Cluster Profiler (3.18.1), while transcription factor enrichment was calculated using TRRUST website (https://www.grnpedia.org/trrust/) (39). Lineage marker gene sets were determined by stratification of cell types per lineage from the mouse gastrulation reference dataset (2) and by using the “findAllMarkers" function in Seurat (40). Single cell deconvolution was done with R package SCDC (v1.1.3) (41), using the two mixed gastrulation samples from the mouse gastrulation reference dataset as reference. TPM values were calculated using tximport.

### 10X scRNA-seq analysis

Reads were processed and aligned using Cell Ranger (v3.0.2) against mm10. Count matrix was imported in Seurat (v4.0.1) (40). Cells were selected which have 2000 to 8000 detected features per cell and a mitochondrial content between 1 and 10% as good quality cells. The cells are normalized per sample using the NormalizeData function in Seurat, using the ‘LogNormalize’ method and a scaling factor of a 1000. The samples are integrated using the ‘fastMNN’ method. The first 50 dimensions from the MNN reduction was used to determine the UMAP reduction and find clusters. These clusters were used in the ‘findAllMarkers’ function to find genes that are differentially expressed between clusters. SingleR (v1.4.1) was used to annotate cell types using the mouse gastrulation dataset reference (2,42). pySCENIC (v 0.11.2) was used to determine regulon activity per cell using normalized count matrix (43). The Seurat function ‘findAllMarkers’ with the ‘wilcox’ test was used to determine differential regulon activity.

### oxWGBS analysis

Using Cutadapt (v3.7), reads were first trimmed based on quality (PHRED < 20). Custom adapters were removed in paired end mode (‘-a AGATCGGAAGAGC -A AAATCAAAAAAAC’) and only reads with a minimum length of 15 bp were kept. Using Bismark (v0.23.1), the trimmed reads were aligned to GENCODE mm10 GRCm38.p6 with a maximal insert size of 500 bp, followed by deduplication and methylation extraction. Using a custom script the CpG counts were merged to one strand and made in to a file format which can be used in methPipe software (44). For DMR analysis methylation calls for replicates were merged together. On average we detected 19 047 561 CpGs with a coverage >5× in merged replicates. To find differentially methylated regions (DMRs), first lowly methylated regions were determined for each sample using the hmr program which uses a hidden Markov model approach, and a differential methylation score for each CpG between samples was calculated using the methdiff program. Using the dmr program, hypo methylated regions and differential CpGs were combined to form DMRs with minimally 2 differential CpGs per DMR.

DMRs were annotated using AnnotatR (v1.16.0) and ChIPseeker (v1.26.2) packages in R (v4.0.5) using UCSC gene feature and CpG island (CpGi) locations. A DMR was considered to be a ‘CpG island associated DMR’ if the DMR was within 4 kb (CpGi shore + shelf) of a CpGi. DMR subsets were made using bedtools intersect (v2.30.0). Genes were associated with DMRs using the rGREAT (v1.22.0) package. A DMR was considered to be associated with a bivalent domain (which contains both a H3K27me3 and H3K4me3 peak in mouse ESCs) if it was within 3 kb. GO term enrichment was performed using Cluster Profiler (v3.18.1).

5hmC rate was calculated by subtracting oxBS methylation rate (true 5mC) from regular BS methylation rate (5mC + 5hmC) for CpGs which in both datasets had at least a coverage of 5×, while for 5mC CpGs with a 5× coverage in oxBS dataset were used. Profile plots were made using DeepTools (v3.4.3).

### ATAC-seq analysis

ATAC-seq reads were analysed using ENCODE ATAC-seq pipeline (http://doi.org/10.5281/zenodo.156534) developed by Anshul Kundaje's laboratory, which performs quality and adapter trimming, alignment, deduplication, peak calling and quality control in a fully automated manner. The resulting peak files and alignment files were used by DiffBind (v3.0.15) to find differentially accessible regions (DARs) using settings specific for ATAC-seq; ‘summits = 150’ in peak defining and ‘background = true’ in read normalization. Transcription factor motif enrichment in DARs was determined using HOMER (v2) package with default settings. Profile plots and heatmaps were made using DeepTools (v3.4.3) from bigwig files generated from merged replicate reads with the ATAC-seq pipeline.

### CUT&RUN analysis

CUT&RUN reads were first trimmed on quality (PHRED > 20) and length (>20 bp) using TrimGalore! in paired-end mode. Trimmed reads were aligned to GENCODE mm10 GRCm38.p6 using bowtie with specific settings ‘ --local --very-sensitive-local --no-unal --no-mixed --no-discordant -I 10 -X 700’, specifically for CUT&RUN (34), and deduplicated with Picard MarkDuplicates (v2.23.0) using default settings. The duplication rate was < 1%. SEACR was used to call peaks, using stringent mode and normalized against IgG R (v4.0.5) (45). Diffbind (v3.0.15) was used to define differentially enriched regions using default settings. Genes were associated with differentially enriched H3K27me3 regions using the rGREAT (v1.22.0) package. For visualization, bam files of merged replicates were made into bigwig files using BamCoverage (v3.4.3) with settings ‘ --binSize 20 --smoothLength 60 --minMappingQuality 3 --normalizeUsing BPM’. Profile plots and heatmaps were made uinsg DeepTools (v3.4.3).

### ChIP-seq analysis

All public ChIP-seq datasets were analysed in the same way. Khoueiry *et al.* EpiLC TET1 ChIP-seq dataset was downloaded from ArrayExpress under accession E-MTAB-5562 (15). Xiang *et al.* E7.5 tissue H3K27ac ChIP-seq and ATAC-seq, and Cruz-Molina *et al.* ESC ChIP-seq datasets were downloaded from GEO with accession number GSE125318 and GSE89211, respectively (26,46). ENCODE H3K27ac ChIP-seq datasets were directly downloaded directly https://www.encodeproject.org/ as bigwig files without any further processing.

Reads were first quality trimmed (PHRED < 20) and adapters were removed using TrimGalore! (v.0.6.4) with default settings, followed by alignment to GENCODE mm10 GRCm38.p6 with Bowtie2 (v2.3.5.1) and deduplication with Picard MarkDuplicates (v2.23.0) using default settings. For visualization, bam files of merged replicates were made into bigwig files using BamCoverage (v3.4.3) with settings ‘ --binSize 20 --smoothLength 60 --minMappingQuality 3 --extendReads 147 (not for PE files) --normalizeUsing BPM’. Profile plots and heatmaps were made using DeepTools (v3.4.3).

H3K27ac peaks were called using MACS2 (v2.2.7.1) with settings ‘-g mm -B -q 0.01 --nomodel --broad --broad-cutoff 0.1’. Replicate peaks were merged using IDR (v2.0.4.2). H3K27ac were considered distal when the distance to nearest transcription start site (TSS) (based on ENSEMBL version 98) was >3 kb. Intersections were made using bedtools intersect (v2.30.0). Genes were associated with distal regulatory regions by using rGREAT (v1.22.0) and assigned to the enhancer that was nearest to the gene.

Gene promoters were defined by merging TSS from all transcripts in ENSEMBL that overlapped ± 1.5 kb (a total of 3 kb around one TSS), resulting in a count of 74840 promoters, of which 17930 (24%) are associated with a CpGi, determined using AnnotatR (v1.16.0). Based on overlap with H3K4me3 and H3K27me3 peaks from ESC ChIP-seq datasets from Cruz-Molina *et al.* (using the same parameters to call peaks (26), we defined 3594 bivalent promoters, of which 3272 (91%) are associated with a CpGi. Of all CpGi promoters, 3272 are bivalent (18%).

## RESULTS

### Divergent lineage trajectories of *Tet1*^+/+^ and *Tet1*^−/−^ cells over a differentiation time-course

**Figure 1.**
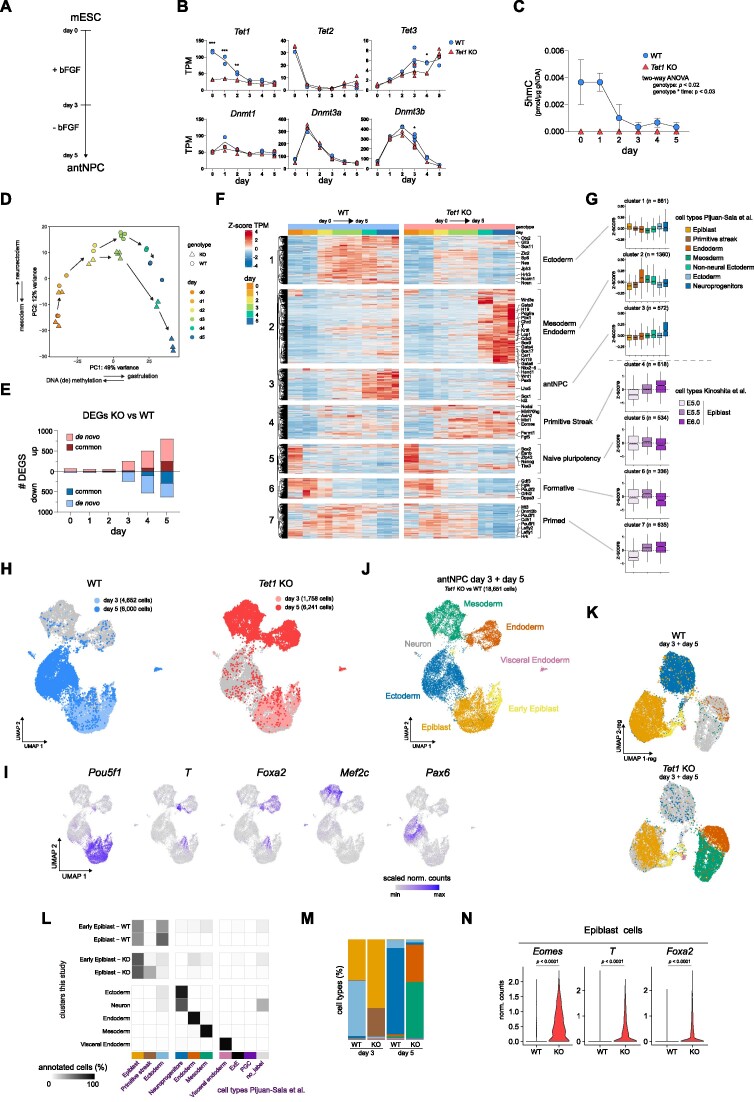
Divergent lineage trajectories of *Tet1*^+/+^ and *Tet1*^−/−^ cells over a differentiation time-course. (**A**) Schematic of basic fibroblast growth factor (bFGF)-driven neurobasal differentiation protocol to convert mouse ESCs into anterior neural progenitor cells (antNPCs) in 5 days. (**B**) Expression levels of TET DNA dioxygenases and DNA methyltransferases (DNMTs) during differentiation of wild type (WT) and *Tet1* KO ESCs in transcripts per million (TPM) measured using bulk RNAseq. Data are shown as the mean of four samples at day 3, three samples at day 5 and otherwise 2 samples at all other time-points. ***P-value < 0.001; **P-value < 0.01; *P-value < 0.05. (**C**) Quantification of 5hmC based on dot blots. Data are shown as mean ± SEM of *n* = 3 independent ESC lines per genotype. (**D**) PCA plot of bulk RNA-seq data obtained during the differentiation time course. Representative terms and direction of GO processes that are enriched in top 100 genes contributing to each principal component are indicated next to the axes. (**E**) Number of up and down-regulated differential expressed genes (DEGs) in *Tet1* KO versus WT per day, classified as *de novo* or in common with DEGs observed earlier. DEGs are defined based on a *P* adjusted value <0.05 and log_2_ |fold change| > 1. (**F**) Time course heatmap of a composite of DEGs based on genotype pairwise comparison and temporal analysis, clustered using k-means clustering and shown as the *Z*-score of TPM values. (**G**) *Z*-score based on log-normalized counts or TPM of cluster genes in Figure 1F as measured in different collated lineages from scRNA-seq reference dataset (2), or E5.0, E5.5 and E6.0 bulk RNA-seq datasets (7). (**H**) UMAP of integrated scRNA-seq data of both *Tet1* KO and WT cells collected at day 3 and day 5 of differentiation. For clarity the UMAP is split by genotype. (**I**) Expression levels of lineage markers projected on UMAP space and expressed as scaled normalized counts. (**J**) Lineage identity of UMAP clusters. (**K**) UMAP based on regulon activity in day 3 and day 5 scRNA-seq samples, shown separately per genotype. Regulons are coloured to correspond to their lineage identity shown in (J). (**L**) The percentage of cells annotated to collated cell types from the mouse gastrulation scRNA-seq reference dataset (2) per defined cluster as defined in (J). Since the Early Epiblast and Epiblast clusters are composed of roughly equal proportion of both WT and *Tet1* KO cells from day 3, they are split per genotype. (**M**) Proportion of annotated cell types per sample, using the colour scheme per cell type shown in (L). (**N**) Expression of primitive streak markers *Nanog*, *T* and *Foxa2* in *Tet1* KO and WT cells only in the Epiblast cluster, shown as normalized counts.

As an *in vitro* model for neural induction, we used a well-established serum-free protocol to convert mouse ESCs into monolayer cultures of anterior neural progenitor cells (antNPCs) in 5 days (26,27) (Figure 1A). This ‘reductionist’ monolayer differentiation model was chosen (over cellular aggregation into embryoid bodies or gastruloids) to achieve better reproducibility and homogeneity of neural cell generation with anterior-dorsal identity, which can enhance the robustness of downstream epigenome analyses. We previously characterized the differentiation of 4 independent littermate pairs of *Tet1*^+/+^ (wild type, WT) and *Tet1*^−/−^ (knockout, KO) ESC lines (all male) derived from (B6 × 129S6)F1-*Tet1*^tm1Koh^ mouse blastocysts under ‘neurobasal differentiation’ conditions, in which basic fibroblast growth factor (bFGF) is supplemented during the first 72 hours in neurobasal N2B27 defined media without further exogenous signalling inhibition (16). In that previous study, we observed efficient induction of antNPC gene markers (*Pax6*, *Sox1* and *Lhx5*) in WT cells on day 5 and significant loss of expression in *Tet1* KO cells across all replicates (16). In this study, we examined 2 pairs of WT and *Tet1* KO ESC lines (see Methods for strain characterization) further by collecting cells at daily intervals over the 5-day neurobasal differentiation time-course for RNA-sequencing (RNA-seq). Expression of *Tet1* and *Tet2* in WT cells diminished rapidly within the first 2 days (Figure 1B, Supplementary Figure S1A, B). *Tet3* expression increased marginally by day 5, but remained lowly expressed, and 5hmC diminished to undetectable levels by the third day, suggesting a dearth of TET activity during the last 3 days of differentiation (Figure 1B, C, Supplementary Figure S1B, C). Expression of *Dnmt3a* and *Dnmt3b* genes encoding *de novo* DNA methyltransferases peaked on day 1 and 2–3, respectively, in both WT and *Tet1* KO cells (Figure 1B), suggesting that *Tet1* KO cells are likely to accrue DNA hypermethylation by day 2.

Principal component analysis (PCA) of the transcriptome datasets showed that *Tet1* KO cells diverged from WT cells starting on day 3 and were clearly separated by day 5 on principal component 2 (PC2) (Figure 1D). On the PC2 axis, the top 100 genes were enriched for gene ontology (GO) terms associated with neuroectoderm, while the bottom 100 were enriched for terms associated with mesoderm development, reflecting the distinct lineage fates of WT and *Tet1* KO cells (Supplementary Table S2). Using a stringent threshold (FDR adjusted *P*-value < 0.05 and log_2_ |fold change| > 1) to find biologically significant differentially expressed genes (DEGs) between *Tet1* KO versus WT per day, we observed roughly equal numbers of up-regulated (1359) and down-regulated (1074) DEGs starting on day 3 (Figure 1E). The majority of DEGs detected on days 4 (>80%) and 5 (>60%) were *de novo*, suggesting highly dynamic transcriptomic changes as differentiation progressed. By combining TET1-dependent DEGs together with temporal DEGs defined over multiple time points (total of 4967 unique genes) and then performing k-means clustering, we defined seven clusters based on the composite DEGs (Figure 1F). Other than classic marker genes, we used several datasets, consisting of single cell RNA-seq (scRNA-seq) obtained from gastrulating E6.5–E8.5 mouse embryos (2), bulk RNA-seq from E5.0, E5.5, and 6.0 mouse epiblasts (7), and mouse ESCs and antNPCs (26), to annotate each cluster (Figure 1G, Supplementary Figure S1D). Clusters 5, 6 and 7, consisting of genes associated with the naive (*Essrb*, *Zfp42*), formative (*Pou2f2*) and primed (*Dnmt3b*) pluripotency states respectively (8), were sequentially down-regulated over the first 3 days with comparable dynamics in both *Tet1* KO and WT cells (Figure 1F, G). The transcriptome profiles positioned formative epiblast-like cells (EpiLCs, see Materials and Methods), an *in vitro* correlate of the E5.5–6.0 pre-streak stage epiblast (28,29), and post-implantation epiblast-derived stem cells (EpiSCs), resembling the E7.5 anterior primitive streak (47), at day 1 (Supplementary Figure S1E) and day 2–3, respectively, in the differentiation trajectory. Cluster 1 (enriched for early ectoderm markers including *Otx2*, *Ncam1, Zic2*) and Cluster 3 (enriched for antNPC markers *Pax6*, *Sox1*, *Lhx5*) were activated sequentially on day 2 and day 4, respectively, in WT but induction was severely compromised in *Tet1* KO cells. Conversely, cluster 2 (enriched for mesoderm/endoderm markers *Nkx2-5*, *Gata4*, *Sox17*, *Pdgfra*) and cluster 4 (primitive streak, e.g. *Eomes*, *Mixl1*) genes were lowly expressed in WT cells but ectopically induced in *Tet1* KO cells between day 3 and 4 (Figure 1F, G).

Further, we benchmarked our bulk gene expression time-course data against marker gene sets obtained from collated cell types and lineages in the reference mouse gastrulation scRNA-seq dataset (2) (Supplementary Table S3), confirming a transient upregulation of primitive streak genes on day 3, endoderm and mesoderm genes on day 4, and downregulation of ectoderm genes from day 2 in *Tet1* KO relative to WT cells (Supplementary Figure S1F). These drastic differences in germ layer cell proportions between *Tet1* KO and WT cells during *in vitro* differentiation were also recapitulated when we deconvoluted our bulk RNA-seq time-course data into different lineages based on the mouse gastrulation reference (2) (Supplementary Figure S1G, H).

To address definitively the extent of cellular heterogeneity in our differentiation cultures, we performed scRNA-seq of single cells collected on day 3 and day 5, which our bulk RNA-seq data suggest to be the time-points of gastrulation onset and completion, respectively. On day 3, *Tet1* KO cells clustered together in one major cluster with WT cells, which we identified to be primed epiblast cells expressing EpiSC markers (*Pou5f1*, *Dnmt3b*, *Pim2*) (Figure 1H, Supplementary Figure S1I, J). By day 5, however, *Tet1* KO cells aggregated as two major clusters expressing lineage markers for mesoderm (*Twist1, Mef2c, Myl7*) and endoderm (*Foxa2, Spink1, Sox17*); these were radically distinct from a single major cluster constituted by WT counterparts expressing ectoderm (*Pax6, Sox1, Sox2*) and neural (*Tubb3*, *Onecut2*) genes (Figure 1H–J, Supplementary Figure S1I, J). A small cluster (<1%) common to both genotypes was identified as visceral endoderm (*Lama1*, *Sox17*) (Supplementary Figure S1I, K). Otherwise, only <4% of day 5 *Tet1* KO cells were found in the ectoderm cluster and vice versa, <3% of day 5 WT cells in the mesoderm and endoderm clusters, indicating a nearly complete lineage identity switch in the absence of TET1. The cluster identities were verified in a UMAP projection based on transcription factor (TF)-based gene-regulatory network-modules, also known as regulons, defined using pySCENIC (43). Here again, *Tet1* KO cells at day 5 showed mainly mesoderm and endodermal transcriptional programs, whereas WT cells showed mainly an ectodermal program (Figure 1K, Supplementary Figure S1L, M).

When we annotated our *in vitro* scRNA-seq datasets using the mouse-gastrulation reference, we confirmed that cells at day 5 scored as post-gastrulation E8.5 cell types (Figure 1L, M, Supplementary Figure S1N). The ectoderm cluster (consisting of WT cells) scored mainly as ‘neuroprogenitor’ cell type, while the endoderm and mesoderm cluster (*Tet1* KO cells) scored as expected ‘endoderm’ and ‘mesoderm’ (Figure 1L). Although day 3 WT and *Tet1* KO clustered together as epiblast cells on UMAP plots, the more precise annotation using the gastrulation reference distinguished a more mature day 3 WT subpopulation as ‘ectoderm’, whereas a more mature day 3 *Tet1* KO subpopulation scored as ‘primitive streak’ (Figure 1L, M). In agreement, expression of core primitive streak genes (*Eomes*, *T, Foxa2*) was already elevated in a significant fraction of *Tet1* KO cells in the Epiblast cluster by day 3 (Figure 1N).

We note that pre-existing *Tet2* expression in ESCs and low basal *Tet3* expression in this differentiation model may compensate for *Tet1* loss-of-function, since the complete absence of TET proteins in triple KO ESCs will severely compromise differentiation (9,48). Nonetheless, our study reveals a dominant role of TET1 at the first lineage bifurcation event of germ layer segregation that determines either neuroectoderm or primitive streak fate.

### Activation of Wnt/β-catenin signalling by de-repression of the *Tcf7l1* gene-regulatory network in *Tet1*^−/−^ cells

**Figure 2.**
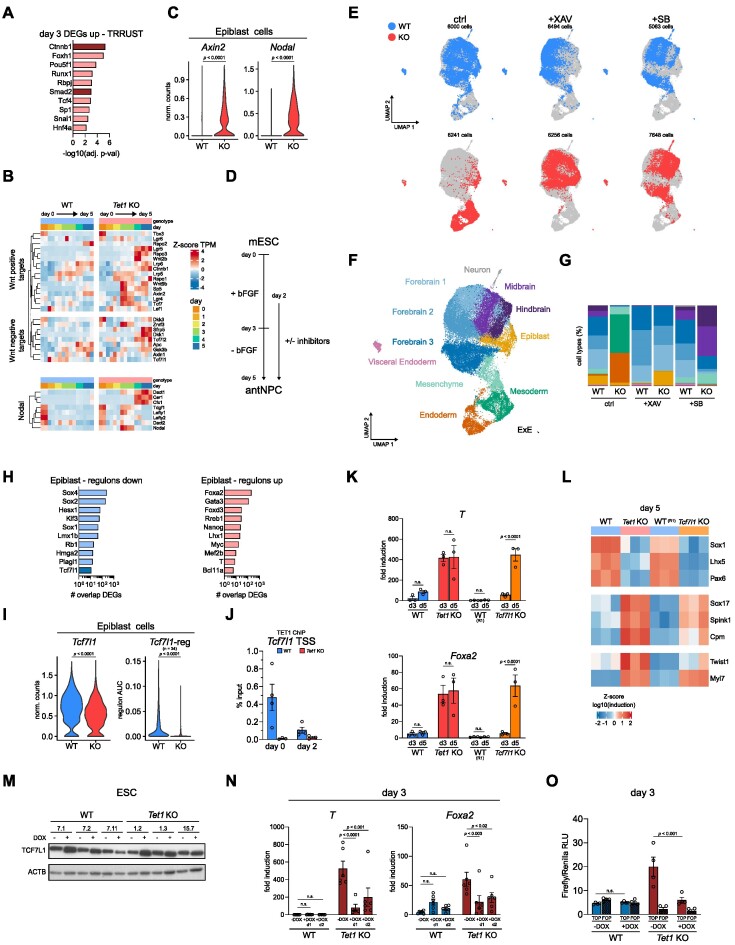
Activation of Wnt/β-catenin signalling by de-repression of the *Tcf7l1* gene-regulatory network in *Tet1*^−/−^ cells. (**A**) Top 10 transcription factors (TFs) predicted to regulate up-regulated DEGs in *Tet1* KO cells at day 3. (**B**) Time-course expression heatmap of genes involved in Wnt positive and negative regulation and Nodal signalling, shown as the *Z*-score of RNA-seq TPM values. (**C**) Expression of direct targets of Wnt (*Axin2*) and Nodal signalling (*Nodal*) in the Epiblast cluster, shown as normalized counts. (**D**) Schematic of directed differentiation towards antNPCs with signalling inhibitors added from day 2. (**E**) UMAP of integrated scRNA-seq samples collected at day 5. WT and *Tet1* KO cells are either untreated or treated with 5 μM of the Wnt inhibitor XAV939 (XAV) or 2.5 μM of the Nodal signalling inhibitor SB431542 (SB). For clarity the UMAP is split per genotype and treatment. (**F**) UMAP clusters coloured by lineage identity. (**G**) Proportion of cell types annotated by lineage cluster per sample, using the colour scheme per lineage cluster shown in (F). (**H**) Selected top 10 differential regulons in *Tet1* KO vs WT cells in Epiblast cluster ranked based on number of overlapping bulk RNAseq collated DEGs. (**I**) Expression level and regulon activity (number of genes in the regulon is indicated within the parenthesis) of *Tcf7l1* in Epiblast cluster cells. (**J**) ChIP qPCR analysis of TET1 binding at *Tcf7l1* transcription start site (TSS) at day 0 and day 2 of differentiation. Data are shown as mean ± SEM of *n* = 4 biological replicates, from two independent differentiations using two different ESC lines per genotype. (**K**) Gene expression of primitive streak markers *T* and *Foxa2* at day 3 and day 5 of neurobasal differentiation (without inhibitor treatment) from *Tet1* KO versus WT ESCs and *Tcf7l1* KO vs WT R1 ESCs with a similar genetic background, measured using qPCR. Data are shown as mean ± SEM of *n* = 3 biological replicates from independent differentiations using two ESC lines of *Tet1* KO and WT cells, and one ESC line of *Tcf7l1* KO and R1 cells. (**L**) Heatmap of qPCR gene expression data of ectoderm (*Sox1*, *Lhx5*, *Pax6*), endoderm (*Sox17*, *Spink1*, *Cpm*) and mesoderm (*Twist1*, *Myl7*) lineage markers in *Tet1* KO and *Tcf7l1* KO ESCs vs their respective controls. Data are shown as *Z*-score of log_10_ transformed fold induction. (**M**) Western blot for TCF7L1 in serum + LIF-cultured ESCs treated for 48 h with 2 μg/ml doxycycline (DOX) to over express *Tcf7l1* in *Tet1* KO and WT stably transfected over-expression clonal lines. (**N**) Expression of primitive streak markers *T* and *Foxa2* at day 3 of differentiation (without inhibitor treatment) from *Tet1* KO and WT ESC lines overexpressing *Tcf7l1*. 2 μg/ml of DOX was added at day 1 or day 2. Data are shown as mean ± SEM of *n* = 6 biological replicates from two independent differentiations using three different ESC lines per genotype. (**O**) Wnt activity based on a transient transfection TOP/FOP-flash reporter assay measured at day 3 of differentiation in *Tet1* KO and WT ESCs, treated with 2 μg/ml DOX from day 1 to over-express *Tcf7l1*. TOP-Flash contains a minimal fos-promoter coupled to Tcf-binding sites upstream of a luciferase reporter; FOP-Flash contains mutated Tcf-binding sites. Data are shown as mean ± SEM of *n* = 4 biological replicates using three different ESC lines per genotype.

Core regulatory networks driving transcriptomic changes may be identified through an interrogation of TF binding motifs in promoters of DEGs using the TRRUST database (39). From this analysis of *Tet1* KO versus WT cells at day 3, TFs associated with the top scoring motifs included *Ctnnb1*, which encodes β-catenin in canonical Wnt-signalling, and *Smad2*, a key TGF-β/Nodal signalling transducer (Figure 2A). Other factors identified were lineage-specific TFs, which were either lowly expressed (*Hnf4a*, *Runx1*), expressed later during differentiation (*Snai1*, *Foxh1*, *Tcf4*), or were not differentially expressed (*Pou5f1*, *Rbpj*, *Sp1*) (data not shown). In line with an activation of Wnt/β-catenin and Nodal signalling, Wnt positive regulators (*Axin2*, *Sp5*, *Tcf7*, *Lgr4*, *Wnt9b*) and Nodal signalling targets (*Nodal*, *Tdgf1*, *Lefty1*, *Lefty2*) were collectively activated in bulk *Tet1* KO cells (Figure 2B) and in single *Tet1* KO epiblast cells (Figure 2C) on day 3. By performing transient transfection of a Wnt reporter construct, we confirmed a 2–3-fold increase in Wnt signalling activity in *Tet1* KO cells on day 3, which was completely suppressed by a Wnt inhibitor XAV939, but not by the Nodal/TGF-β receptor inhibitor SB431542 (Supplementary Figure S2A). Using phosphorylation of SMAD2 as an indicator of Nodal activity, we observed that both XAV939 and SB431542 effectively blocked ectopic Nodal activation in *Tet1* KO cells on day 3, confirming that Wnt/β-catenin acts upstream of Nodal in the signalling cascade (Supplementary Figure S2B).

In our previous study using an embryoid body differentiation model, we have shown that treating *Tet1* KO cells with Wnt and Nodal signalling inhibitors can rescue neuroectodermal fate (16). To assess cellular heterogeneity and anterior-posterior identity, we performed scRNA-seq in *Tet1* KO and WT cells on day 5 after treatment with XAV and SB from day 2 (Figure 2D). In this directed differentiation protocol, both Wnt and Nodal inhibition reverted *Tet1* KO cells from mesoderm and endoderm identity towards neuroectoderm, insomuch that >81% of treated *Tet1* KO cells distributed in one of six ectodermal clusters (forebrain 1, forebrain 2, forebrain 3, midbrain, hindbrain and neuron) (Figure 2E–G, Supplementary Figure S2C–G). Consistent with the posteriorizing effect of Wnt signalling (27), Wnt inhibition by XAV directed >81–84% of *Tet1* KO and WT cells to differentiate towards forebrain identity (*Pax6*, *Rax*, *Lhx5*, *Six3*); in contrast, SB treatment resulted in many more (62%) *Tet1* KO ectoderm cells (compared to only 16% in WT cells) retaining posterior midbrain (*Wnt1*, *Otx2*, *En1*, *En2*) and hindbrain (*En1*, *En2*, *Irx2*, *Pax3*, *Gbx2*, *Fgf8*) identity (Figure 2F, G, Supplementary Figure S2E–G), in line with the presence of residual Wnt activity in these cells (Supplementary Figure S2A). Inhibiting bone morphogenetic protein (BMP) signalling by LDN193189 treatment from day 2 did not affect differentiation (Supplementary Figure S2H), as previously shown in murine cells (49), indicating that hyperactive Nodal signalling via phosphorylation of SMAD2 causes the loss of neuroectoderm in *Tet1* KO, but not hyperactive BMP signalling via SMAD1/5/8.

To identify regulators of Wnt pathway which are targets of TET1, we examined differential regulon activities between *Tet1* KO versus WT specifically in the Epiblast cell cluster. We selected the top 40 up- and 40 down-regulated regulons, and then ordered them by the number of overlapping DEGs (Figure 2H, showing the top 10). Among lineage TFs, we observed as expected up-regulation of classic primitive streak regulons (*Foxa2*, *Nanog, T*) and down-regulation of classic ectoderm regulons (*Sox2*, *Sox1*) in KO compared to WT (Supplementary Figure S2I). The majority of lineage TFs were differentially expressed later in differentiation (after day 3), leading our attention to *Tcf7l1*, which was expressed early during differentiation, and showed differential expression and a significantly down-regulated regulon in the KO Epiblast single-cell cluster (Figure 2H). *Tcf7l1*, also known as *Tcf3* and a canonical direct Wnt repressor in mouse ESCs, has a role in naive to primed pluripotency transition but is also required for lineage specification and mesoderm differentiation (50,51). Consistent with a role in repressing Wnt signalling in WT cells, *Tcf7l1* was downregulated early within days 1–3 in bulk *Tet1* KO cells and expressed at lower levels in single epiblast cells on day 3 (Figure 2I, Supplementary Figure S2J). Suggesting a direct gene regulation, the promotor of *Tcf7l1* was bound by TET1 in both ESCs, EpiLCs, and until day 2 in this neurobasal differentiation assay (Figure 2J, Supplementary Figure S2K). To demonstrate that WT cells can overcome cell intrinsic repression of Wnt/β-catenin pathway, we treated WT cells with a canonical Wnt signalling activator CHIR99021 (CHIR) and observed induction of TOP-flash activity on day 3 by up to + 30-fold; similarly, *Tet1* KO cells responded to CHIR with a further + 5-fold elevation (+20-fold compared to WT without CHIR) in TOP-flash activity (Supplementary Figure S2L). The sensitivity of both WT and KO cells to exogenous Wnt activators suggests that a cell-intrinsic de-repression of signalling transduction contributes to the 2–3 fold elevated basal Wnt/β-catenin activity in KO cells on day 3, rather than ectopic hyperactivation.

Next, we asked whether loss of TCF7L1 in mouse ESCs will result in a lineage switch similar to that observed with the loss of TET1. We used established mouse blastocyst-derived *Tcf7l1* KO cells (24) and subjected them to the neurobasal differentiation assay (with signalling inhibition). As reported previously (24), we observed a delay in exit from pluripotency and differentiation in *Tcf7l1* KO cells, reflected by a persistence in expression of the naive pluripotency marker *Zfp42* on day 1 and lack of expression of differentiation markers on day 3 as detected by quantitative RT-PCR (qPCR) (Figure 2K, Supplementary Figure S2M). However, by day 5 primitive streak markers *T* and *Foxa2* were induced in *Tcf7l1* KO cells at similar levels as in *Tet1* KO cells (Figure 2K). Furthermore, we observed similar extents of mesoderm (*Twist1*, *Myl7*) and endoderm (*Sox17*, *Spink1*, *Cpm*) gene induction and neuroectoderm (*Sox1*, *Lhx5*, *Pax6*) loss in *Tcf7l1* KO and *Tet1* KO cells by day 5 of differentiation (Figure 2L). These results suggest that a TET1-TCF7L1-WNT transcriptional axis may regulate germ layer lineage bifurcation.

To demonstrate whether modulating TCF7L1 levels can affect lineage switching in *Tet1* KO cells, we generated doxycycline (DOX)-inducible *Tcf7l1* over-expression (OE) ESC lines on both WT and *Tet1* KO genotypes. Clonal replicate lines were selected based on inducibility of TCF7L1 protein expression in ESCs upon 48-hour DOX treatment (Figure 2M, Supplementary Figure S2N). We tested addition of DOX on either day 1 or day 2 of neurobasal differentiation and analysed cells at day 3, to determine whether enhancing *Tcf7l1* expression upon pluripotency exit can prevent ectopic primitive streak gene expression in *Tet1* KO cells. Indeed, a 2–4 fold increase in *Tcf7l1* expression by DOX treatment starting on either day 1 or day 2 significantly reduced expression of *T* and *Foxa2* in *Tet1* KO cells, whereby reduction was greater when DOX was added on day 1, while not affecting expression of primed markers *Otx2* and *Dnmt3b* (Figure 2N, Supplementary Figure S2O). In line KO15.7 the induction of TCF7L1 was more variable compared to other lines in differentiation (Supplementary Figure S2O, top line), which correlated with a variable rescuing effect observed in this line (Figure 2N, outliers). Treatment with DOX at day 1 or day 2 of parental *Tet1* KO lines without the TCF7L1 OE construct did not affect primitive streak gene induction (data not shown). Moreover, *Tcf7l1* OE from day 1 significantly reduced the activity of the Wnt reporter in *Tet1 KO* cells (Figure 2O), validating TCF7L1 as a canonical Wnt repressor regulated by TET1 to safeguard against precocious primitive streak fate entry.

### Contribution of 5mC oxidation by TET1 in the repression of primitive streak fate

**Figure 3.**
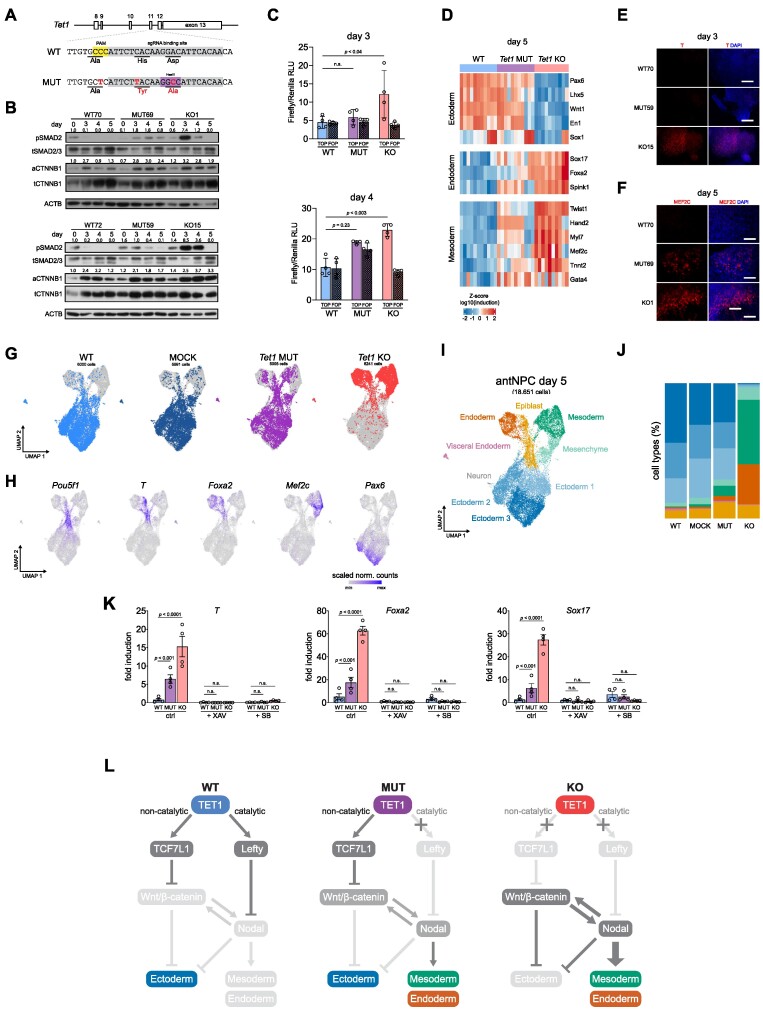
Contribution of 5mC oxidation by TET1 in the repression of primitive streak fate. (**A**) Schematic of CRISPR-Cas9 target sequences to introduce mutations disrupting the catalytic activity of TET1 by homology directed repair. WT and mutant (MUT) sequences encoding the active site are indicated, with altered bases and amino acids in red. Guide RNA sequence is highlighted in grey, PAM site in yellow and the introduced restriction site used for genotyping in purple. (**B**) Western blot for phospho-(p)SMAD2 and active β catenin (aCTNNB1) as readouts of Nodal and Wnt signalling, respectively, at day 0 (ESC), 3, 4 and 5 of neurobasal differentiation (without inhibitors) using *Tet1* KO, *Tet1* mutant (MUT), and mock WT control cells. Two clonal ESC lines were examined per genotype. Bands are quantified and normalized to total protein (top band of tSMAD2/3 or tCTTNB1); normalized expression values are shown above each lane on the blot. (**C**) Wnt activity assay at day 3 and day 4 of differentiation using TOP/FOP-flash reporter. Data are shown as mean ± SEM of n = 4 biological replicates from two clonal ESC lines per genotype. (**D**) Heatmap of qPCR gene expression analysis of ectoderm (*Pax 6*, *Lhx5*, *Wnt1*, *En1*, *Sox1*), endoderm (*Sox17*, *Foxa2*, *Spink1*), and mesoderm (*Twist1*, *Hand2*, *Myl7*, *Mef2c*, *Tnnt2*, *Gata4*) lineage markers at day 5 of neurobasal differentiation. Data are shown as Z-score of log_10_ transformed fold induction, *n* = 8 using 3 clonal ESC lines per genotype in 4 independent differentiations. (E, F) Immunofluorescence staining of primitive streak marker protein T at day 3 (**E**) and mesoderm transcription factor MEF2C at day 5 (**F**) of differentiation. Scale bar indicates 100 μm. (**G**) UMAP of integrated scRNA-seq samples of *Tet1* KO, MUT, mock WT control and WT cells collected at day 5. For clarity cells of each individual sample are coloured separately on the UMAP plots. (**H**) Expression levels of lineage markers projected on UMAP space as scaled normalized counts. (**I**) UMAP clusters at day 5 coloured by lineage identity. **J)** Proportion of cell types annotated by lineage cluster per sample at day 5. Colours correspond to lineage identities shown in (I). (**K**) Gene expression based on qPCR analysis of mesoderm (*T*) and endoderm markers (*Foxa2 and Sox17)* in *Tet1* KO, MUT and WT cells at day 5 following treatment with 5 μM XAV, 2.5 μM SB or DMSO as vehicle control. Data are shown as mean ± SEM of *n* = 4 biological replicates from two clonal ESC lines per genotype. (**L**) Schematic of a bipartite mode of TET1 to regulate lineage bifurcation. In WT cells, both Wnt and Nodal signalling are repressed leading to ectoderm differentiation. In *Tet1* KO, repression of *Tcf7l1* and *Lefty* activates Wnt and Nodal signalling, respectively, leading to mesoderm and endoderm lineage specification. In *Tet1* MUT cells, early activation of Nodal pathway is not sufficient to inhibit neuroectoderm differentiation. However, ‘leaky’ Nodal signalling induces Wnt signalling later in development, leading to all three lineages being present.

Since TET1 has a N-terminal domain with distinct regulatory functions from its C-terminal catalytic domain (52), we asked how lineage bifurcation choice is dependent on its catalytic or non-catalytic function. We used CRISPR/Cas9 and homology directed repair to introduce H1620Y and D1622A substitutions within the Fe^2+^ chelating active site coding sequence in endogenous *Tet1* to disrupt its 5mC oxidation (i.e. catalytic) function (11) (Figure 3A). Three independent pairs of *Tet1* catalytic mutant (MUT) and isogenic mock transfected (MOCK) ESC lines were generated in the (B6 × 129S6)F1 strain (Supplementary Figure S3A). We validated complete loss of 5hmC in *Tet1* MUT lines upon *in vitro* conversion to EpiLCs, a state when *Tet1* is expressed in the absence of *Tet2* and *Tet3*, and during the neurobasal differentiation time course (Supplementary Figure S3B, C). All three *Tet1* MUT ESC lines expressed full-length TET1 protein and down-regulated expression with similar kinetics as MOCK cells during antNPC differentiation (Supplementary Figure S3D-E). ChIP-qPCR analysis at lineage-specific enhancers and promoters also indicated similar binding affinities and dynamics between WT and MUT at the early stages of differentiation (Supplementary Figure S3F).

We and others previously identified the *Lefty1* and *Lefty2* loci, encoding the Nodal antagonist LEFTY, to be direct targets of DNA demethylation by TET1 in mouse ESCs (14–15,53). Sustaining *Lefty* expression prevents premature primitive streak differentiation (54). Here, we verified that a specific loss of TET1 catalytic activity was sufficient to induce an increased and sustained phosphorylation of SMAD2 in *Tet1* MUT cells from day 3 onwards, although the hyperactivation was less compared to *Tet1* KO cells, suggesting that Nodal signalling is at least partially repressed by TET1’s catalytic activity (Figure 3B). The western blot analysis also indicated higher levels of active β-catenin protein at day 5 in *Tet1* MUT and KO cells compared to WT controls (Figure 3B); however, differential levels of active β-catenin protein were not detectable at day 3. By performing the Wnt reporter activity assay, we detected elevated Wnt/β-catenin activity in *Tet1* KO cells on day 3 and day 4, but no significant increase in signalling activity in *Tet1* MUT cells above background at the same time-points (Figure 3C). (Limitations of transient transfection after 2 days of differentiation precluded a readout of the Wnt reporter on day 5.) Thus, Wnt/β-catenin signalling initiation upon TET1 dysfunction appears to require complete loss of TET1 protein. In this differentiation model, stimulating WT cells on day 2 exogenously with the Nodal agonist Activin A can activate Wnt/β-catenin, verifying a cross-talk between Nodal and Wnt/β-catenin signalling (Supplementary Figure S3G). These observations suggest that the specific loss of TET1 catalytic activity first triggers hyperactivation of Nodal/Smad signalling, which subsequently activates Wnt/β-catenin, resulting in a positive feedback loop of Wnt and Nodal pathways amplifying each other.

To understand transcriptomic differences between *Tet1* MUT and KO cells during differentiation, we performed bulk RNA-seq on day 3 and day 5. In PCA plots, *Tet1* MUT cells clustered together with WT cells and away from *Tet1* KO cells, on both day 3 and day 5 (Supplementary Figure S3H). Using the same stringent criteria (FDR adjusted *P*-value < 0.05 and log_2_|fold change| > 1) to define DEGs on day 3 and day 5 between *Tet1* MUT versus WT, we observed only about 50 DEGs on day 3, mostly downregulated in *Tet1* MUT but with no functional enrichment. On day 5, genes up-regulated in *Tet1* MUT were enriched in GO terms associated with mesoderm development, while downregulated genes showed no functional enrichment (Supplementary Figure S3I, J, Supplementary Table S2). These results are similar with those by a recent study that also examined *Tet1* catalytic mutant ESCs and observed normal differentiation towards neuroectoderm, much like WT ESCs (22). Therefore, TET1’s non-catalytic function is dominant in driving neural fate induction.

However, by performing qPCR, we could reliably detect expression of markers of all three lineages in *Tet1* MUT cells on day 5 (ectoderm: *Pax6*, *Sox1*, *Lhx5*, *Wnt1*, *En1*; endoderm: *Sox17*, *Foxa2*, *Spink1*; mesoderm: *Myl7*, *Mef2c*, *Tnnt2*, *Hand2*, *Twist1*, *Gata4*) (Figure 3D). Although primitive streak markers such as T were not detectable in *Tet1* MUT cells on day 3, the mesoderm TF MEF2C was clearly detected in both *Tet1* MUT and *Tet1* KO cells by immunofluorescence on day 5 (Figure 3E,F), reflecting a delayed activation of Wnt/β-catenin and Nodal/Smad signalling in *Tet1* MUT cells during differentiation. These results suggest that *Tet1* MUT cells may have gained tri-lineage potential, losing the restriction that directs WT cells unilaterally towards neuroectoderm and *Tet1* KO cells towards primitive streak fate. To verify the lineage heterogeneity of *Tet1* MUT cells on day 5, we performed scRNA-seq of *Tet1* MUT and MOCK cells. MOCK cells aggregated as one major cluster overlapping fully with WT ectoderm and neuronal cells (expressing *Pax6*, *Sox1*, *Sox2*, *Tubb3*); only <1% clustered together with endoderm (expressing *Foxa2*, *Sox17*) and mesoderm cells (expressing *Mef2c*, *Twist1*) (Figure 3G–J, Supplementary Figure S3K–M). In contrast, *Tet1* MUT cells distributed in all three lineage clusters; while the majority (>70%) aggregated within the ectoderm cluster, the cells also formed distinct clusters expressing endoderm (4%) and mesoderm (8%) lineage markers. By treating the cells with both XAV or SB from day 2 onwards, we could inhibit mesoderm and endoderm marker gene expression completely in MUT cells, as in KO cells, confirming that the alternative fates observed in *Tet1* MUT cells arise from ectopic Wnt and Nodal signalling (Figure 3K).

These results suggest that a bipartite mode of TET1 activities at an early branch point of germ layer lineage bifurcation involves (i) a non-catalytic regulation that initially represses Wnt/β-catenin signalling to promote epiblast transition into neuroectoderm, and (ii) a 5hmC-dependent catalytic regulation that is repressive of Nodal activation and subsequently Wnt signalling, preventing premature entry into primitive streak fate (Figure 3L).

### Preferential engagement of ectodermal enhancers by TET1

**Figure 4.**
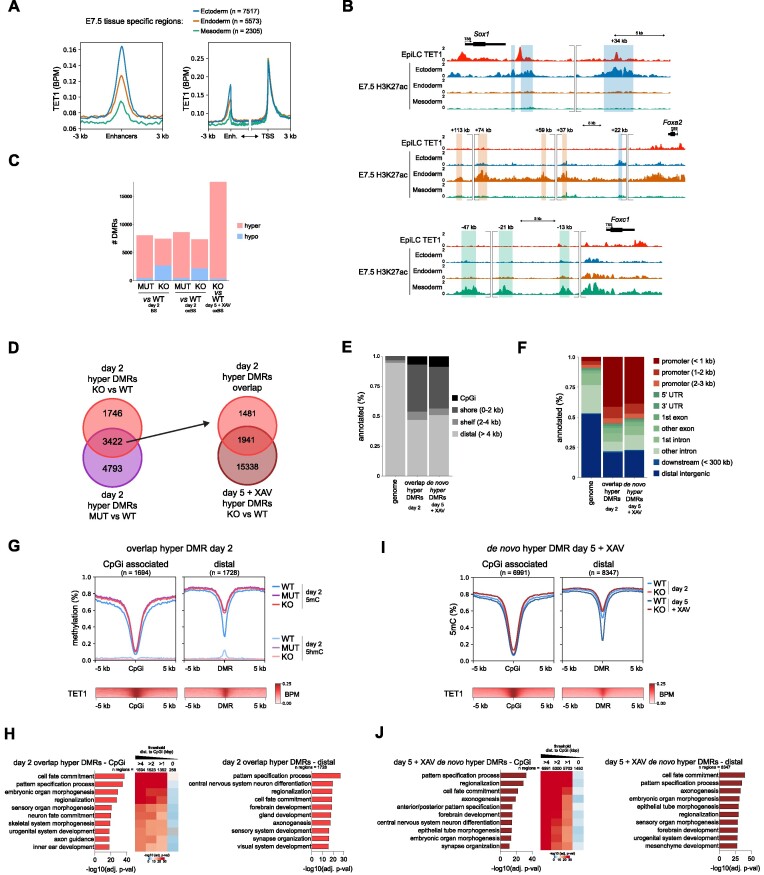
TET1 demethylates CpGi shores and enhancers in primed pluripotency. (**A**) Average profile plots of TET1 genome occupancy, calculated as number of reads per bin/ sum of all reads per bin (in millions) (BPM) in EpiLCs (also now known as formative ESCs) at E7.5 lineage-specific enhancers (left), and linked with TSSs uniquely associated with those enhancers (right; see Materials and Methods). (**B**) Integrated genome viewer (IGV) tracks of TET1 ChIP-seq peaks in EpiLCs and E7.5 tissue-specific H3K27ac ChIP-seq (signal in BPM) at example loci showing enriched TET1 binding at ectoderm specific enhancers (15). Distance to TSS is indicated; tissue specific enhancers are indicated with blue (ectoderm), orange (endoderm) and green (mesoderm) shadings. (**C**) Number of hyper or hypo differentially methylated regions per comparison, *Tet1* KO vs WT or MUT vs WT, detected at day 2 or day 5 (+XAV) of differentiation by performing oxidative (oxBS) or regular bisulfite sequencing (BS). (**D**) Left panel, Venn diagram of the overlap between KO versus WT and MUT versus WT hyper DMRs from the day 2 oxBS dataset by which DMRs in the overlap are considered to be high-confidence sites of TET1-dependent DNA demethylation. Right panel, Venn diagram of the overlap of day 2 hyper DMRs with KO versus WT hyper DMRs from the day 5 (+XAV) oxBS dataset, by which day 5 DMRs not in the overlap are considered to be *de novo* from day 2. (E, F) Genomic features based on CpG island proximity (**E**) and gene structure (**F**) annotation of hyper DMRs subsets at day2 and day 5 (+XAV) compared to background of the entire genome. (**G**) Profile plots of 5mC and 5hmC methylation levels inferred from the combined analysis of BS and oxBS datasets at day 2 in KO, MUT, WT cells, classified as CpGi-associated and distal DMRs. Below, heatmaps of TET1 occupancy in EpiLCs centred at both classes of day 2 DMRs. Note, CpGi-associated DMRs are centred at nearest CpGi while distal DMRs are centred on the DMR itself (26). (**H**) Top 10 GO-terms enriched in genes associated with CpGi-associated (left) or distal (right) DMRs. Heatmap shows enrichment score for GO-terms when different cut-off distances are used to define CpGi-associated hyper DMRs; cut-off of 0 means DMRs overlap with CpGi. (**I**) Profile plots of true 5mC at *de novo* hyper DMRs of day 5 (+XAV) in KO and WT cells, superimposed on 5mC levels of the same regions at day 2. Below, heatmaps of TET1 occupancy in EpiLCs, centred at the respective DMRs (15). (**J**) Top 10 GO-terms enriched in genes associated with CpGi-associated (left) or distal (right) *de novo* DMRs at day 5 (+XAV), similar to H.

Since the distinct regulatory modes of TET1 appear associated with lineage-specific outcomes, we examined whether the genomic occupancy patterns of TET1 may identify lineage-specific determinants. We have previously reported a dynamic repatterning of TET1 genomic occupancy from mainly promoters in naive pluripotency to both promoters and poised enhancers in formative pluripotency (16). By further incorporating a published H3K27ac ChIP-seq dataset that defined active enhancers and promoters in germ layer tissues of the E7.5 mouse embryo (46), we associated TET1-bound loci to genes classified by their individual germ layer tissue-specificity of expression (Supplementary Figure S4A). By this analysis, we observed that TET1 preferentially occupied ectodermal distal enhancers in formative pluripotency (mimicked *in vitro* by EpiLCs), prior to the expression of the associated genes at E7.5 (Figure 4A, B). However, at gene proximal promoter regions uniquely linked to every lineage-specific enhancer, TET1 occupancy was equally distributed among the three lineages (Figure 4A, right panel). Enhancers are often CpG poor, whereas 60–70% of mammalian protein-coding gene promoters are CpG-rich (55,56) and feature CpG islands (defined as stretches of DNA on average 1000 bp long with an observed over expected CpG ratio ≥ 0.6) (57). Therefore, we speculated that these differential CpG densities between gene proximal and distal loci may be intricately linked with the dependence on TET1’s 5mC oxidation activity.

### TET1 demethylates CpGi shores and enhancers in primed pluripotency

Based on the kinetics of TET expression and genomic 5hmC content (Figure 1B, C, Supplementary Figure S1B, C), we anticipated day 2 to be the earliest time-point to display DNA methylome changes specifically due to loss of TET1. Therefore, we performed whole genome bisulfite sequencing (WGBS) in conjunction with oxidative bisulfite (oxBS) conversion (58) to obtain base resolution maps of 5mC and 5hmC in biological replicates of *Tet1* KO, MUT, and WT cells collected on day 2 of differentiation. In both WGBS and oxWGBS datasets, we detected ± 7500–8500 differentially methylated regions (DMRs) in both *Tet1* KO versus WT and MUT versus WT comparisons (Figure 4C). In *Tet1* MUT cells, almost all DMRs (>95%) were hypermethylated (i.e. hyper DMRs); but in KO cells, about 30% were hypomethylated relative to WT (Figure 4C). These hypo DMRs specific to KO cells overlapped with ESC-specific active enhancers and promotors (Supplementary Figure S4B), were bound by TET1 in EpiLCs (Supplementary Figure S4C) and interestingly, displayed gain of 5hmC concomitantly with loss of 5mC (Supplementary Figure S4D), suggesting that they may be *de novo* sites of active DNA demethylation in the complete absence of TET1 protein. As also described by a recent study by Chrysanthou *etal*., the catalytic dead full length TET1 protein may be protecting these *Tet1* KO-specific hypo DMRs from compensatory DNA demethylation by TET2 or TET3 (22).

Hyper DMRs in either *Tet1* KO or MUT cells were enriched in CpG island (CpGi) shores (2-kb long regions flanking CpGi) (59) and gene promoters (Supplementary Figure S4E, F). To focus on DMRs that can be directly attributed to loss of TET1 catalytic function, we selected 3422 hyper DMRs common in KO and MUT cells (i.e. the overlap of hyper DMRs), which recapitulated a roughly equal distribution among CpGi shores and distal regions, for further analysis (Figure 4D-F). These common hyper DMRs exhibited on average a 40% gain in CpG methylation levels compared to WT cells and a width spanning 200–500 bp (Supplementary Figure S4G, and data not shown). We divided these hyper DMRs into two classes: DMRs within 4kb of a CpGi as ‘CpGi-associated’ (*n* = 1694), and those further away as ‘distal’ (*n* = 1728). While 61% of CpGi-associated DMRs were within 1 kb of a CpGi, our threshold distance of 4kb was chosen to encompass also CpGi shores and CpGi shelves, defined to be within 0.2 kb and 2–4 kb, respectively, of a CpGi (59). Using publicly available H3K4me3, H3K27me3, H3K4me1, and H3K27ac ChIP-seq datasets profiled in mouse ESCs (26), we noted that CpGi-associated DMRs displayed hallmarks of promoters marked by active H3K4me3 and repressive H3K27me3 signatures, known as bivalent domains (60), while distal DMRs were enriched for H3K4me1 and intermediate levels of H3K27ac marks, indicative of active or primed enhancers (Supplementary Figure S4I). By this classification, 82% of all genes (total *n* = 1701) associated with CpGi-associated DMRs are within 10 kb of their transcription start sites (TSSs), while only 25% of all genes (total *n* = 1728) associated with CpGi poor distal DMRs are within 10 kb (Supplementary Figure S4J, K).

When CpGi-associated DMRs were centred on the CpGis, we observed that CpGs within CpGi centres were constitutively unmethylated (<10% methylation) in all three genotypes, as expected (Figure 4G, left panel). The methylation gains in CpGi-associated hyper DMRs were found mainly at CpGi flanking regions in *Tet1*-deficient (KO and MUT) cells relative to WT cells, in agreement with this class harbouring mostly CpG shores (Figure 4G, left panel). More striking methylation differences between WT and *Tet1*-deficient cells were seen at the centre of distal DMRs (Figure 4G, right panel). As evidence for TET-mediated DNA demethylation in both DMR classes, 5hmC was detectable only in WT cells at the regions corresponding with methylation gains in KO and MUT cells (Figure 4G). Moreover, both DMR classes centred at TET1 binding peaks in EpiLCs (Figure 4G, bottom panel). They are highly enriched in GO terms associated with pattern specification and embryonic morphogenesis across all germ layers, although distal DMRs appeared to be more specifically enriched for GO terms associated with central nervous system development (Figure 4H). Different CpGi distance thresholds from 1–4 kb did not affect these results (Figure 4H, heatmap).

Next, we asked whether hyper DMRs induced by loss of TET1 early at day 2 would persist to affect neuronal genes later in differentiation. For this, *Tet1* KO and WT ESCs were converted to day 5 antNPCs in the presence of the Wnt inhibitor XAV added at day 2, a treatment that rescues the lineage identity switch in *Tet1* KO and allows both WT and KO cells to form homogeneous antNPCs of predominantly E8.5 forebrain identity (Figure 2E–G). Since 5hmC is undetectable by dot blot analysis in day 5 antNPCs (Figure 1C), we focused on profiling true 5mC changes caused by loss of TET1 by oxWGBS.

We detected 17665 DMRs between *Tet1* KO and WT antNPCs on day 5, of which > 98% are hyper DMRs (Figure 4C). Similar to day 2 hyper DMRs, day 5 DMRs were 150–450 bp wide, showed 40% higher median methylation levels in *Tet1* KO relative to WT, and were enriched at promoter proximal CpGi shores with an equivalent number also found at distal regions (Supplementary Figure S4E, F, H). Of all 17 279 hyper DMRs, 15 338 were *de novo* DMR sites that did not overlap with hyper DMRs on day 2 (Figure 4D). They were distributed among promoter proximal CpGi shores (*n* = 6991) and distal enhancer sites (*n* = 8347) marked by H3K4me3 and H3K4me1, respectively, and displayed moderate levels of H3K27ac in ESCs to indicate that they were active regulatory sites prior to differentiation (Figure 4E, F, Supplementary Figure S4L–N). Methylation differences were more apparent at distal sites, where further DNA demethylation (>40% reduction in 5mC levels) occurred in WT but not in KO antNPCs on day 5 compared to day 2 (Figure 4I, right). At CpGi-associated regions, *de novo* DMRs showed a 10–15% reduction in 5mC levels at CpGi flanking regions in WT antNPCs on day 5, but hypermethylation in KO at CpGi centres (Figure 4I, left). Of note, TET1 protein was barely detectable in WT cells between day 3 and day 5 of differentiation (Supplementary Figure S1B) but was engaged at *de novo* DMRs (both CpGi-associated and distal) in EpiLCs (day 1) prior to neural fate induction (Figure 4I, bottom panel), suggesting that the presence of TET1 at pre-gastrulation safeguards against ectopic DNA hypermethylation post-gastrulation. Day 5 *de novo* hyper DMRs were associated with genes that are enriched in GO terms associated with organogenesis including ‘pattern specification process’, ‘axonogenesis’, and ‘forebrain development’ (Figure 4J).

Since many developmental genes are described to be located within long (>5 kb) hypomethylated regions called DNA methylation valleys (DMVs) or canyons (61–63), we asked whether TET1 activity regulates these regions. Using the WT methylome, we defined 566 DMVs on day 2 of differentiation, and 698 DMVs on day 5 (+XAV treatment). In congruence with the profiles of CpGi-associated DMRs, DMVs were affected (10%) in both *Tet1* KO and MUT cells on day 2 mainly at their boundaries where 5hmC was detectable in WT cells (Supplementary Figure S4O). On day 5, increase of methylation at DMV boundaries was as much as 10–15% at both boundaries and valleys (Supplementary Figure S4P). These results suggest a DMV protective role for TET1.

### Non-catalytic regulation of chromatin accessibility by TET1 at distal enhancers at gastrulation onset

**Figure 5.**
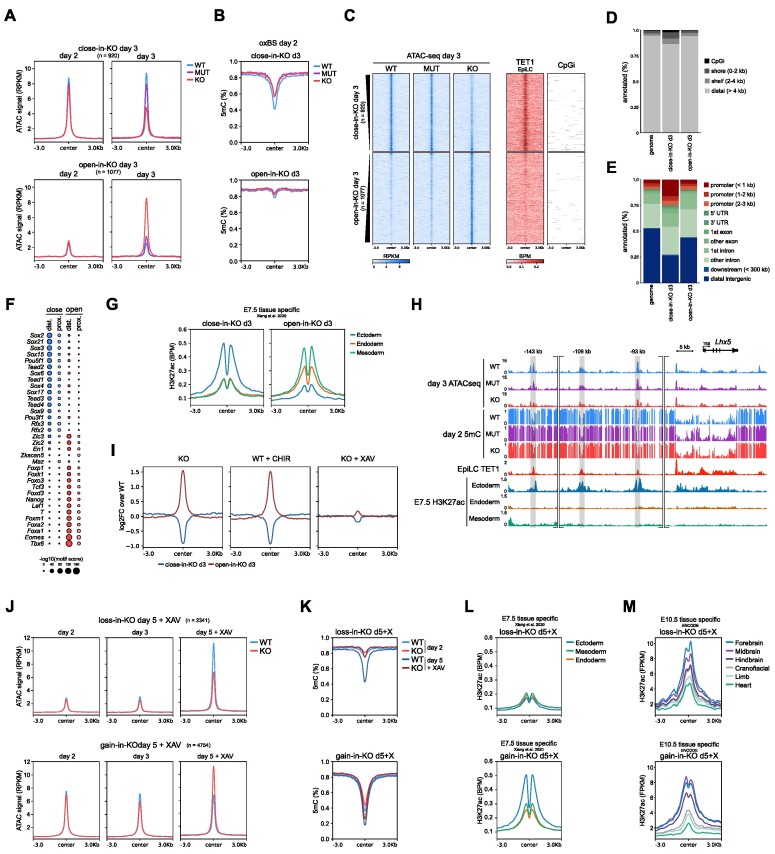
Non-catalytical regulation of chromatin accessibility by TET1 at distal enhancers. (**A**) Profiles of ATAC-seq signal in reads per kilobase million (RPKM) centred at day 3 close-in-KO (top panels) or open-in-KO (bottom panels) differentially accessible regions (DARs) at day 2 (left) and day 3 (right) of neurobasal differentiation in *Tet1* KO, MUT and WT cells. (**B**) Profiles of 5mC levels at day 2, centred at day 3 DARs. (**C**) Heatmaps of ATAC-seq signal in RPKM in KO, MUT and WT cells at day 3 of differentiation, TET1 binding in BPM in EpiLCs, and CpGi frequency centred at day 3 close-in-KO (top panels) and open-in-KO (bottom panels) DARs ordered by fold change of differential accessibility. (D, E) Genomic features based on CpG island proximity (**D**) and gene structure (**E**) annotation of close-in-KO and open-in-KO DARs at day 3. (**F**) Scores for top 20 close-in-KO and open-in-KO transcription factor (TF) motifs in distal (close-in-KO d3, *n* = 625, 68%; open-in-KO d3, *n* = 902, 84%) or proximal (close-in-KO d3, *n* = 295, 32%; open-in-KO d3, *n* = 175, 16%) DARs, filtered by a minimum TF expression of 5 TPM on at least one time-point during the RNA-seq time-course. A DAR was considered to be distal if the distance to TSS was >3 kb. (**G**) Profiles of H3K27ac ChIP-seq in BPM in E7.5 germ layer-specific tissues (Xiang *et al.*, 2020) centred at day 3 close-in-KO and open-in-KO DARs (46). (**H**) IGV tracks for ATAC-seq signal at day 3, 5mC levels at day 2, TET1 binding in EpiLCs and E7.5 germ layer-specific H3K27ac ChIP-seq signal at three *Lhx5* distal enhancers. Close-in-KO DARs are highlighted in grey. (**I**) Log_2_ fold changes of ATAC-seq signal in KO cells at day 3, WT cells treated for 24 h with 3 μM of the Wnt activator CHIR99021 (CHIR), and KO cells treated for 24 h with the Wnt inhibitor XAV939 (XAV), compared to day 3 WT cells, centred at day 3 close-in-KO and open-in-KO DARs. (**J**) Profiles of ATAC-seq signal in RPKM centred at day 5 (+XAV) loss-in-KO (top panels) or gain-in-KO (bottom panels) differentially accessible regions (DARs) at day 2 (left), day 3 (middle), and day 5 (+XAV) (right) of differentiation in *Tet1* KO and WT cells. (**K**) Profiles of true 5mC levels at day 2 and day 5 (+XAV), centred at day 5 (+XAV) DARs. (**L**) Profiles of H3K27ac ChIP-seq in BPM in E7.5 germ layer-specific tissues (Xiang *et al.*, 2020) centred at day 5 (+XAV) loss-in-KO and gain-in-KO DARs (46). (**M**) Profiles of H3K27ac ChIP-seq in FPKM in ENCODE E10.5 mouse tissues (31), centred at day 5 (+XAV) DARs.

For further insights into the impact of TET1’s catalytic and non-catalytic activities on the chromatin state, we investigated the chromatin accessibility changes in WT, *Tet1* MUT and KO cells during differentiation using the assay for transposase accessible chromatin-sequencing (ATAC-seq). On day 2, we did not find any significant differential accessible regions (DARs) between *Tet1* KO or MUT versus WT, suggesting that TET1-dependent DNA methylation changes at day 2 precede any opening and closing of chromatin that would drive lineage segregation (Supplementary Figure S5A). However, by day 3, *Tet1* KO cells clearly diverged from MUT and WT cells on the PCA (Supplementary Figure S5A). We detected 1997 DARs (920 close, 1077 open) between KO versus WT and only 4 DARs (2 open, 2 close) between MUT versus WT (Supplementary Figure S5B), suggesting that DARs result primarily from loss of non-catalytic TET1 activity. The regions that closed in *Tet1* KO on day 3 (close-in-KO) were regions already accessible on day 2 in all three genotypes; interestingly, these regions still exhibited DNA hypermethylation in *Tet1* MUT cells on day 2 despite sustaining an open state, illustrating a discordance between DNA methylation changes and chromatin accessibility (Figure 5A, B). In contrast, regions that opened (open-in-KO)on day 3 were *de novo* accessible regions previously highly methylated in all genotypes on day 2 (Figure 5A, B). Only close-in-KO DARs were bound by TET1 in EpiLCs (Figure 5C); thus, the engagement of TET1 protein at these regions, independently of its catalytic function, is required to sustain an open chromatin state. On the other hand, DARs that opened were bound very weakly or not at all by TET1, suggesting that chromatin opening at these regions is an indirect effect of loss of TET1 (Figure 5C).

Both open-in-KO and close-in-KO DARs were devoid of CpGi and CpG poor (Figure 5C, Supplementary Figure S5C); close-in-KO DARs even showed near-zero CpG frequencies, consistent with their inability to engage TET1 whose CXXC domain confers an affinity for CpG-rich loci (64). Only < 25% of close-in-KO and < 10% of open-in-KO DARs can be annotated as promotors, while the vast majority were annotated as distal (Figure 5D, E), marked by H3K4me1 instead of H3K4me3 (Supplementary Figure S5D). From these data, we classified the DARs as distal regulatory regions.

To determine how chromatin accessibility at DARs are regulated by lineage TFs, we performed motif enrichment analysis on DARs that were classified either as distal (close-in-KO d3, *n* = 625, 68%; open-in-KO d3, *n* = 902, 84%) or proximal (close-in-KO d3, *n* = 295, 32%; open-in-KO d3, *n* = 175, 16%). (A DAR was considered to be distal if the distance to TSS was >3 kb.) To find biological significant TFs, we filtered our list based on gene expression, including only TFs that were expressed at a minimal TPM of 5 at one point during antNPC differentiation in either *Tet1* KO or WT cells (Supplementary Figure S5E, unfiltered list). Close-in-KO DARs enriched for motifs belonging to ectoderm-specific TFs that harbour high mobility group (HMG) (*Sox2*, *Sox3*), homeobox (*Pou5f1*) and TAE domains (*Tead1*). In contrast, open-in-KO DARs enriched for mesoderm- and endoderm-specific TFs with forkhead (*Foxa2*, *Foxo3*) and T-box protein motifs (*T*, *Eomes*) (Figure 5F). Proximal regions were enriched for similar motifs as distal regions, although distal regions had a higher enrichment score correlating with their relative higher abundance. Interestingly, Wnt signalling factor *Lef1* was highly enriched in opened DARs, while not in closed DARs (Figure 5F). By utilizing H3K27ac ChIP-seq and ATAC-seq datasets profiled in E7.5 germ layer tissues (46), we verified that close-in-KO and open-in-KO DARs are respectively ectoderm- and mesoderm/endoderm-specific lineage enhancers (Figure 5G, H, Supplementary Figure S5F).

Since modulation of Wnt signalling can alter lineage choice, we asked whether the chromatin state of *Tet1*-dependent DARs would also be responsive to Wnt/β-catenin signalling activity. To answer this, we performed ATAC-seq on day 3, after a 24-hour treatment of either WT cells with the Wnt signalling activator CHIR or *Tet1* KO cells with the Wnt inhibitor XAV. Simulating loss of TET1 but with more extensive chromatin accessibility changes, CHIR-treated WT cells diverged from WT and *Tet1* MUT in similar directions as *Tet1* KO cells on the PC1 axis, whereas XAV-treated *Tet1* KO cells reverted to cluster together with WT and *Tet1* MUT (Supplementary Figure S5G). We observed that the extent of chromatin accessibility changes at both close-in-KO and open-in-KO DARs can be fully mimicked by treatment with the Wnt agonist in WT cells, whereas all changes can be reverted to WT state in KO cells by Wnt inhibition (Figure 5I, Supplementary Figure S5H), suggesting that the DARs are target loci of Wnt/β-catenin signalling.

To further address the question whether aberrant methylation in post-gastrulation forebrain cells affects the chromatin state, we performed ATAC-seq of day 5 antNPCs treated with XAV from day 2. We detected 2341 DARs that showed loss of accessibility in *Tet1* KO on day 5 (+XAV) compared to WT (loss-in-KO d5 + X), and 4754 regions that gained accessibility (gain-in-KO d5 + X). Loss-in-KO d5 + X DARs were *de novo* accessible regions, where accessibility was still very low on day 2 or day 3; in contrast, gain-in-KO d5 + X were already accessible on day 2 (Figure 5J). Interestingly, loss-in-KO d5 + X DARs, which showed high methylation levels in both WT and *Tet1* KO on day 2, showed loss of methylation in WT cells but not in *Tet1* KO on day 5, suggesting that the persistence of high DNA methylation levels at these loci on day 5 is associated with reduced chromatin opening in KO (Figure 5J,K). Both types of DARs were classified as distal enhancers based on histone marks, CpG frequencies, and occupancy by TET1 in EpiLCs (Supplementary Figure S5I-K). Gain-in-KO d5 + X DARs were enriched in H3K27ac in E7.5 ectoderm tissue relative to mesoderm/endoderm (46), consistent with these being ‘neural default’ loci that are already accessible in the epiblast (Figure 5L). Loss-in-KO d5 + X regions were not strongly enriched in any E7.5 tissues; however, by utilizing ENCODE tissue-specific H3K27ac ChIP-seq datasets, we found that these DARs were highly enriched for H3K27ac in more differentiated E10.5 fetal brain tissues (Figure 5L, M) (31,46). The latter observations suggest that the absence of TET1 causes *de novo* accessible fetal brain enhancer loci to exhibit a persistently high DNA methylation status coupled with compromised chromatin accessibility post-gastrulation, prior to gene expression later in development.

These results suggest that a dominant role of TET1 in ESCs is to maintain open chromatin at ectodermal-specific distal enhancers until gastrulation onset (on day 3 of differentiation), which promotes ‘neural default’ differentiation and indirectly keeps mesoderm and endoderm enhancer loci closed from Wnt/β-catenin signals driving alternative fates. This early function at distal loci does not require its catalytic activity and can occur independently of DNA demethylation. However, the opening of chromatin post-gastrulation at fetal brain distal enhancers is coupled with DNA demethylation.

### Functional interactions between TET1 and Polycomb at bivalent gene promoters

**Figure 6.**
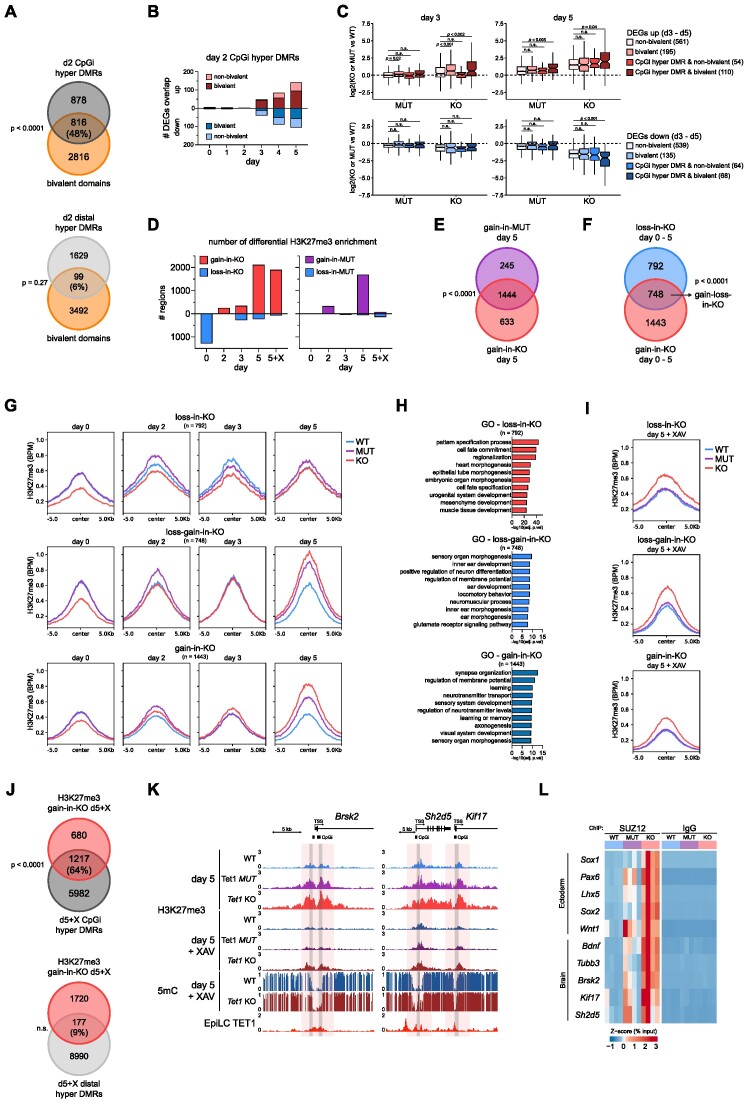
Functional interactions between TET1 and Polycomb at bivalent gene promoters. (**A**) Venn diagram of the association between bivalent domains and high-confidence TET1-dependent hyper DMRs at day 2, classified as CpGi-associated and distal DMRs. (**B**) Number of bulk RNA-seq DEGs (KO vs WT) per day of neurobasal differentiation (without inhibitors) that are within 10 kb of day 2 TET1-dependent hyper DMRs stratified based on bivalency and presence of a CpGi-associated hyper DMR. (**C**) Change in gene expression at day 3 or day 5 in *Tet1* KO or MUT cells compared to WT cells for all unique DEGs collated from day 3 until day 5, stratified based on bivalency and presence of a TET1-dependent and CpGi-associated hyper DMR. (**D**) Number of differential H3K27me3 CUT&RUN peaks per day in KO versus WT and MUT versus WT comparisons. Day 5 samples were examined with (+X) or without XAV treatment. (**E**) Venn diagram of regions that gained H3K27me3 in *Tet1* KO and MUT at day 5 of differentiation (without XAV) compared to WT. (**F**) Venn diagram of H3K27me3 loss-in-KO and gain-in-KO regions collated from day 0 to day 5. The overlap is described as H3K27me3 loss-gain-in-KO. (**G**) Profile plots of H3K27me3 CUT&RUN signal in BPM at loss-in-KO (top), loss-gain-in-KO (middle), and gain-in-KO regions (bottom) during the differentiation time-course in *Tet1* KO, MUT, and WT cells. (**H**) Top 10 GO terms for genes associated with loss-in-KO, loss-gain-in-KO and gain-in-KO regions. (**I**) Profile plot of H3K27me3 CUT&RUN signal in BPM at day 5 (+XAV) loss-in-KO, loss-gain-in-KO and gain-in-KO. (**J**) Venn diagrams of H3K27me3 gain-in-KO regions and either CpGi-associated or distal hyper DMRs at day 5 (+XAV). (**K**) IGV tracks for H3K27me3 CUT&RUN signal in BPM at day 5 (±XAV), 5mC levels at day 5 (+XAV), and TET1 binding in EpiLCs at *Brsk2*, *Sh2d5* and *Kif17*. Differential H3K27me3 gain-in-KO regions at day 5 (with or without XAV) are indicated in red, KO versus WT day 5 (+XAV) hyper DMRs are highlighted in grey. (**L**) Heatmap of SUZ12 ChIP-qPCR at loci where there is a significant increase of H3K27me3 in *Tet1* KO and MUT at day 5, plotted as -log_10_(% input). Data are shown as mean ± SEM of *n* = 4, using two independent lines per genotype and 2 technical ChIP replicates per line. In the Venn overlap analyses of A, E and J, all promoters in the genome (*n* = 74 840) were used as background for a hypergeometric test.

Several studies have described co-localization of 5hmC with histone bivalent domains, where H3K27me3 marks are mediated by PRC2, in mouse and human ESCs (18,20,21,65,66). However, the functional impact of 5hmC in the context of histone bivalency on gene expression remains unclear. Because CpG methylation can reduce the binding affinity of PRC2 components to nucleosomes (67), we asked whether loss of 5mC oxidation may disrupt Polycomb-mediated developmental gene repression. Interestingly, a significant portion (48%) of day 2 hyper DMRs overlapped with ‘bivalent’ gene promoters (Figure 6A). (The expected rate would be ± 18%, the percentage of all CpGi promoters that are bivalent in the mouse genome.) In contrast, only 6% of distal hyper DMRs overlapped. By identifying genes within 10 kb of a CpGi-associated hyper DMR (total n = 1259) that were DEGs of KO versus WT from day 3 onwards during neurobasal differentiation (without signalling inhibitors, which revealed the full extent of lineage segregation, Figure 1D), we observed that more than 66% of up-regulated DEGs (130 of 197) were genes with bivalent promotors in ESCs (Figure 6B).

To determine the functional effect of DNA hyper methylation at bivalent domains on gene expression, we stratified all up- and down-regulated DEGs in *Tet1* KO or MUT versus WT based on whether they were associated with a CpGi hyper DMR and/or a bivalent promoter (Figure 6C), then compared the fold changes (FC) in expression of both *Tet1* KO and MUT over WT cells on day 3 and day 5 of neurobasal differentiation. On day 3, we observed a significant activation among bivalent DEGs (with or without CpGi-associated hyper DMRs) already in *Tet1* KO cells, although no significant gene expression changes were detectable in MUT cells. However, by day 5, bivalent DEGs with CpGi-associated hyper DMRs showed significantly greater fold increase in gene expression compared to other DEG classes in both KO and MUT cells (average log_2_FC > 2 and 1, respectively) (Figure 6C, top panels). Such positive correlations between DNA methylation gains at promoter-proximal CpGi shores and gene expression were evident at primitive streak bivalent genes such as *T* and *Sox17* (Supplementary Figure S6A). On the other hand, bivalent DEGs with CpGi-associated hyper DMRs also showed significantly greater fold decrease in expression at day 5, but only in KO cells since much fewer DEGs were down-regulated in MUT cells (Figure 6C, bottom panels and S3J). Thus, a functional impact of TET1’s catalytic activity on lineage segregation appears restricted to CpGi-associated promoter regions, where a synergy with its non-catalytic activity suppresses alternative non-neural fates.

To determine how loss of TET1 affects Polycomb function before and after lineage segregation, we performed Cleavage Under Target & Release Using Nuclease (CUT&RUN) to profile genome-scale changes in H3K27me3 in WT, MUT and KO cells during the differentiation time-course. On the PCA, *Tet1* KO cells clustered away from MUT and WT cells on day 0, while on day 2 and day 3 all three genotypes clustered together. However, both MUT and KO cells clustered separately from WT cells by day 5 without inhibitor treatment (Supplementary Figure S6B). These dynamics were reflected by the differential peak analysis. On day 0, we observed a loss of H3K27me3 in KO, but not in MUT cells, relative to WT; however, the loss in KO recovered by day 2 after which both KO and MUT made dramatic gains of H3K27me3 by day 5 (Figure 6D). The majority of H3K27me3 gain-in-KO on day 5 overlapped with the gain-in-MUT regions, even though only > 15% of cells in MUT were mesoderm or endoderm compared to > 95% in KO, suggesting that the gain of H3K27me3 occurred independently of cell lineage identity (Figure 6E, 3J). Further, the majority (87%) of differential H3K27me3 regions lost in KO occurred on day 0, while most regions that gained H3K27me3 on day 2 and day 3 retained the gain relative to WT until day 5 (Supplementary Figure S6C, D). To assess whether the gain in H3K27me3 is dependent on the signalling modulation, we treated cells from day 2 onwards with XAV to obtain a homogeneous forebrain population in all three genotypes. Here, KO cells still gained H3K27me3 on day 5, despite the complete conversion to antNPCs. In contrast, MUT cells no longer gained H3K27me3 (Figure 6D), suggesting that the post-gastrulation H3K27me3 gain resulting from loss of TET1 catalytic activity is dependent on Wnt/β-catenin (and downstream Nodal) signalling.

To understand the functional gene categories affected by the dynamic loss and gain of H3K27me3, we classified the differential regions into three groups (Figure 6F, G): (i) regions that lost H3K27me3 in KO on day 0, and recovered by day 2 without subsequent gain (loss-in-KO, *n* = 792); (ii) regions that lost H3K27me3 in KO on day 0 and then subsequently gained the mark by day 5 (loss-gain-in-KO, *n* = 748); and (iii) regions that were not significantly differential on day 0 but gained H3K27me3 after (gain-in-KO, *n* = 1443). All three groups showed bivalent histone marks in ESCs (26), occupancy by TET1 in EpiLCs (15) and similar CpG frequencies comparable with those of promoters (Supplementary Figure S6E–G). H3K27me3 loss-in-KO regions were enriched for mesoderm and ectoderm genes; in contrast both loss-gain-in-KO and gain-in-KO regions are associated with ectoderm and brain-specific genes (Figure 6H). With XAV treatment, all three groups gained H3K27me3 in KO cells, but not in MUT cells, at day 5 (Figure 6I). These H3K27me3 gain-in-KO regions (day 5 + XAV) overlapped significantly with CpGi-associated hyper DMRs detected under the same conditions (Figure 6J), suggesting that DNA hypermethylation and increased H3K27me3 converge at neuronal gene promoters post-gastrulation in the absence of TET1. This was especially apparent at genes with known functions in the brain such as *Brsk2*, *Sh2d5* and *Kif17* (Figure 6K) (68–70).

To confirm that the gain of H3K27me3 observed is associated with increased PRC2 engagement, we performed ChIP-qPCR of SUZ12, a PRC2 core-component, at 5 ectoderm- and 5 brain-specific genes that showed increased H3K27me3 in both *Tet1* KO and MUT at day 5 (without signalling inhibition). This verified a strong increase of SUZ12 binding in KO compared to WT cells, and also a weaker but detectable increase in MUT cells (Figure 6L). Altogether, these data suggest that a predominantly non-catalytic function of TET1 in ESCs facilitates co-operation with PRC2 to repress primitive steak genes until gastrulation onset, consistent with previous reports of TET1 recruiting PRC2 to these genes in ESCs independently of DNA demethylation (22,23). However, the cooperative interaction is rapidly superseded by an antagonistic interaction at neuroectodermal promoters post-lineage priming (after day 2). Though still predominantly an effect of non-catalytic TET1 activity, the repression of developmental signalling by TET1 catalytic function also contributes toward repelling Polycomb from neuronal targets.

### Early developmental origin of disease-associated DMRs in *Tet1*^−/−^ mice.

Treatment with the Wnt inhibitor XAV from day 2 onwards resulted in a homogeneous population of forebrain cells in both WT and *Tet1* KO cells, despite the accumulation of many hyper DMRs over regulatory regions in KO, suggesting that DNA hypermethylation caused by loss of TET1 *per se* does not inhibit neural induction in the absence of alternative fate-inducing signalling cues. Further, many of the genes associated with the DMRs were lowly expressed in day 5 WT antNPCs (Supplementary Figure S7A), suggesting a latent effect of promoter DNA hyper methylation on gene expression. To address whether these genes would be expressed later during development, we used the ENCODE mouse development dataset, which contains bulk RNA-seq data of 16 different tissues at eight different time-points during mouse embryo development ranging from E10.5 to P0 (31). Of all 6193 genes associated with *de novo* hyper DMRs, we kept 4980 genes that had an expression level of minimally 10 TPM in at least one tissue at one time-point to select genes that are expressed and biologically relevant during development. By this analysis, we observed that expression of these genes increased specifically in the ectoderm tissues such as forebrain, midbrain, hindbrain, and neural tube after E15.5, but either did not change or decrease in expression in endodermal and mesodermal tissues (Supplementary Figure S7B).

**Figure 7.**
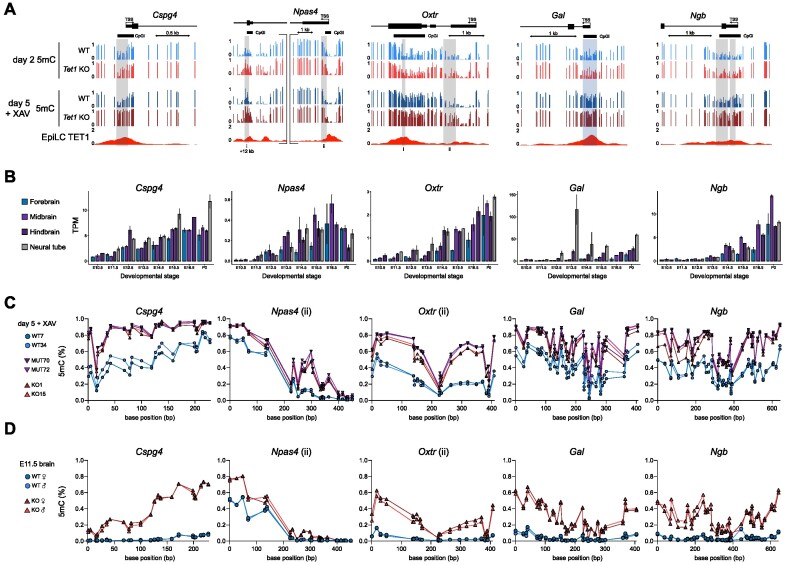
Early developmental origin of disease-associated DMRs in *Tet1*^−/−^ mice. (**A**) IGV tracks of 5mC methylation levels in KO and WT cells at day 2 and day 5 (+XAV), and TET1 occupancy in EpiLCs, at 5 genes known to be hypermethylated in adult *Tet1* KO mice and implicated in neural dysfunction: *Cspg4*, *Gal*, and *Ngb* (Cheng *et al.*), *Npas4* (Rudenko *et al.*) and *Oxtr* (Williams *et al.*). DMRs are highlighted grey. At *Gal*, a region of interest bound by TET1, though not a DMR, is highlighted in blue. (**B**) RNA-seq expression in TPM of indicated genes in specific tissues per developmental stage from ENCODE (31), shown as mean ± SEM of *n* = 2 samples per condition. (C, D) 5mC levels at hyper DMRs at the promoters of *Cspg4*, *Gal*, *Ngb*, *Npas4* (region ii), and *Oxtr* (region ii) in *Tet1* KO, MUT and WT (**C**) day 5 (+XAV) antNPCs and (**D**) E11.5 brains from sex and stage-matched littermates, measured using amplicon bisulfite sequencing.

Previous work by the groups of Xu, Dawlaty and Jiang (71–73) demonstrated that adult *Tet1* KO mice that survived embryonic development were impaired in hippocampal neurogenesis, cognition and memory function, synaptic plasticity, and social and maternal care behaviours. Because TET1 expression is detectable in the adult mouse brain, these phenotypes have been attributed to the loss of active DNA demethylation by TET1 at neuronal gene loci in post-mitotic neurons in the adult brain, supporting an important role for TET1’s catalytic function in neurological disorders. To determine whether aberrant DNA methylation at neuronal genes affected in adult *Tet1* KO mice can already be detected during gestation, we selected 5 neuronal TET1 target genes (*Cspg4*, *Npas4*, *Oxtr*, *Gal* and *Ngb*) that showed DNA hypermethylation in the adult KO mouse brain in the aforementioned studies. The CpGi promoter regions of these five genes showed strong TET1 binding peaks in EpiLCs, low methylation in both WT and *Tet1* KO cells at day 2 of differentiation, but pronounced gain in 5mC to high levels in day 5 *Tet1* KO antNPCs (XAV treated) relative to WT controls (Figure 7A). Interestingly, these genes were not expressed during different stages of pluripotency, neural induction or NPCs (TPM < 1 in ESCs or antNPCs at day 5 + XAV, data not shown). In the ENCODE mouse development datasets, they were only expressed later in development in WT mice (>E12.5) and in the post-natal mouse brain (>P0) (Figure 7B), in agreement with their suggested role in neurological functions later in life (31,71–73).

Using targeted bisulfite sequencing, we confirmed that these loci were hyper methylated in both *Tet1* KO and MUT cells (Figure 7B, C). To further validate that they are hyper methylated early in development *in vivo*, we collected E8.5 anterior neural (headfold) tissues (Supplementary Figure S7D) and E11.5 embryonic brains (Supplementary Figure S7E) from sex- and stage-matched *Tet1* KO and WT littermate individual embryos and performed targeted bisulfite sequencing. (Because of material scarcity, only three loci were analysed in E8.5 headfolds.) The methylation profiles of all analysed loci at both developmental stages showed hypermethylation in *Tet1* KO with similar contours as observed in day 5 (+XAV) antNPCs *in vitro* (Figure 7D, Supplementary Figure S7F), suggesting that the DMRs persist after gastrulation and are not erased by TET2 and TET3 later in development. These results support the notion that hyper DMRs affecting these brain-specific genes associated with a neurodegenerative phenotype in adult mice, may originate as early as post-gastrulation as a result of catalytic dysfunction of TET1.

## DISCUSSION

A dual function of TET1 in gene regulation has been previously described, by which 5mC oxidation activity at promoters and enhancers is thought to promote transcriptional activation while the repressive function is a result of 5mC oxidation-independent interactions with co-repressors (15,17–19). Further, activation of putative lineage enhancers is proposed to be preceded by DNA demethylation, although causal evidence in embryonic development is lacking (9). Here, our results provide fresh insights into the dual function by demonstrating (i) a dynamic switch from a cooperative TET1 interaction with PRC2 at primitive streak genes pre-gastrulation to an antagonistic effect during differentiation, when TET1’s catalytic activity participate in repelling PRC2 from neuronal genes via the control of developmental signalling; (ii) an uncoupling between DNA demethylation and the maintenance of chromatin accessibility at neuroectoderm enhancers at gastrulation onset (day 3). Despite a preferential engagement by TET1 at primed neuroectodermal enhancers, 5mC oxidation at CpG-poor distal sites, or the lack of, has barely any impact on enhancer function. Rather, the engagement of full-length catalytically dead TET1 suffices to maintain chromatin accessibility to promote neuroectodermal fate entry. A functional impact of TET1’s catalytic activity becomes more apparent post-lineage priming, when a synergy with its non-catalytic activity restricted to CpGi-associated promoter regions serves primarily to suppress alternative fates. We identified the Wnt repressor TCF7L1 as an early target of TET1 as well as longer-term effects of TET1’s catalytic activity that sustains demethylation at several loci associated with adult-onset neurodegeneration phenotypes.

Our earlier work has alluded to a dominant role of non-catalytic TET1 function during peri-gastrulation development in the mouse (15). Recently two studies were published on distinguishing the catalytic and non-catalytic functions of TET1 in mouse ESCs (22,23). Much in agreement with our findings, both studies showed that catalytic mutation of *Tet1* alone did not recapitulate the transcriptomic phenotype observed in *Tet1* KO ESCs, despite observing common hypermethylation signatures in both MUT and KO cells. Chrysanthou *et al.* showed that loss of TET1 resulted in dysregulation of bivalent genes by disrupting recruitment of EZH2 and SIN3A and H3K27me3 deposition, but the effects were not dependent on the catalytic activity of TET1 because the bivalent genes were not associated with DMRs. Stolz *et al.* focused on the non-catalytic role of TET1 in the repression of endogenous retroviral elements via the establishment of H3K9me3 and H4K20me3, showing also a decrease in H3K27me3 on a global scale in *Tet1* KO but not in MUT ESCs. However, we found a subset of hyper DMRs that occurred in both MUT and KO cells at CpGi shores, of which ±50% were in the proximity of bivalent domains and associated with dysregulated genes. Further, the loss of H3K27me3 in *Tet1* KO ESCs was restored within 2 days of differentiation. Thereafter, H3K27me3 increased at ectodermal targets by day 5, independently of lineage-directing signalling modulation in KO, but by a Wnt-signalling sensitive mechanism in MUT. These differences between our study and those prior can be readily attributed to the distinct developmental time points assessed. Both Chrysanthou *et al.* and Stolz *et al.* limited their analyses to mouse ESCs cultured under serum and LIF conditions, a naive metastable state that sustains *Tet2* expression. We have performed oxWGBS at day 2 of differentiation in cells that have reached primed state, when *Tet1* is the only TET gene with detectable expression, and a time-course analysis of H3K27me3 occupancy throughout differentiation to a post-gastrulation stage. The developmental time-points are critical because we have observed highly dynamic changes in TET1 genome occupancy and 5mC/5hmC patterns when converting naive mouse ESCs into formative EpiLCs (15,16). Thus, we expect the analysis of DMRs in day 2 cultures (equivalent to a stage of lineage priming), together with the impact on differential enrichment of H3K27me3 until day 5 (post-gastrulation) in this study, to reveal *de novo* TET1-regulated sites that have functionally relevant impact on developmental gene expression.

Histone bivalency, first observed at ESC promoters marked by both active H3K4me3 and repressive H3K27me3 marks, is a hallmark of stem and progenitor cells, but its exact function remains a matter of debate (60). It is widely accepted that bivalency ‘primes’ developmental genes for faster activation or silencing via resolution into monovalent states during lineage segregation. This concept also extends to distal regulatory elements, where the co-presence of H3K4me1 and H3K27me3 histone modifications at ‘poised’ enhancers marks their associated genes for activation later in development (26). However, a recent study proposed that bivalent domains may function instead to prevent the premature activation of genes during germ layer differentiation, thus having a ‘reining’ effect as opposed to ‘priming’ (74). In that study, loss of the 5mC sensitive CpGi-binding protein BEND3 led to a reduction of H3K27me3 in ESCs and a premature activation of developmental genes later during differentiation. Here, our results appear consistent with an interaction of TET1 with a ‘reining’ function of Polycomb-mediated bivalency, at least during pluripotency and early differentiation. Intriguingly, bivalent hyper DMRs in *Tet1* MUT cells are associated with up-regulated expression (predominantly mesoderm genes) by day 5, suggesting that active DNA demethylation by TET1 may also contribute to Polycomb repression of non-neural genes during neural induction. In contrast, both catalytic and non-catalytic TET1 activities contribute to preventing an ectopic gain of Polycomb repressive histone methylation at ectodermal genes post-gastrulation. In this functional antagonism between TET1 and Polycomb during neural induction, TET1 may well be regulating a ‘priming’ effect of histone bivalency. How an ectopic H3K27me3 gain affects subsequent activation of gene targets, the presence of H3K4me3 and occupancy by Trithorax group proteins (75–77), as well as interactions with other epigenetic priming factors such as DPPA2 and DPPA4 (78,79), will be interesting questions to resolve to discern clearly between ‘reining’ versus ‘priming’ effects of TET1’s interaction with histone bivalency, and the possibility of their dependence on genomic and lineage context. Nonetheless, both catalytic and non-catalytic regulation by TET1 are involved in the timely resolution of histone bivalency during lineage segregation, in agreement with the colocalization of TET1 and 5hmC with bivalent gene promoters in ESCs (18,20,21,65,66,80).

The subset of hyper DMRs associated with CpGi shores also reflects TET1 function at DMV boundaries (61–63). DMVs are associated with a majority of developmental genes and are enriched for the Polycomb-deposited H3K27me3. Interestingly, they can display gain in DNA methylation upon gene activation in a similar fashion as we observed at bivalent genes associated with CpGi associated hyper DMRs (61). The Polycomb complex has a strong affinity for unmethylated CpGs and may regulate hypomethylation of DMVs through recruitment of TET1 (20,67,81). Conversely, CpG methylation reduces the binding affinity of PRC2 components to nucleosomes and can therefore disrupt Polycomb function at bivalent domains (67). Indeed, it was recently revealed that QSER1 interacts with TET1 in DMVs to safeguard developmental programs from *de novo* methylation by DNMT3 (82). Although collective evidence, including this study, suggests that functional interactions between TET1 and PRC2 are predominantly independent of TET1 catalytic activity in ESCs (17,20,22,80), a contribution of 5mC oxidation by TET1 at bivalent domains in DMVs to stabilize PRC2 association is not mutually exclusive from non-catalytic interactions.

A direct effect of TET1 catalytic activity in repressing premature primitive steak gene expression reconciles with previous findings by us and others demonstrating the Nodal antagonist *Lefty* genes as direct targets of TET (14–16,53). Although *Tet1* MUT ESCs differentiate predominantly into antNPCs much like WT ESCs, we could detect enhanced SMAD2 phosphorylation in both *Tet1* MUT and KO bulk differentiation cultures. Collectively, these observations causally link TET1 catalytic activity at *Lefty* loci with antagonism of Nodal signalling. Yet, we observed a dominance of Wnt/β-catenin over Nodal signalling in driving cell fate changes in *Tet1* KO cells. In solid cancer lines, loss of TET enzymes and 5hmC has been linked to reduced expression of Wnt repressors due to promoter DNA hyper methylation, resulting in augmented Wnt/β-catenin signalling and enhanced epithelial-mesenchymal transition (83,84). We discovered loss of the Wnt repressor *Tcf7l1* and its regulatory network activity early during differentiation of *Tet1* KO cells, as a cell-intrinsic trigger of mesoderm skewing which can be rescued by over expression of TCF7L1. However, we did not find TET1-dependent DMRs at direct Wnt pathway regulators. Neither did we detect early Wnt/β-catenin signalling activity, Wnt-associated target gene expression or chromatin changes in *Tet1* MUT cells at day 3 as observed in KO cells, suggesting that the triggering mechanism for elevated Wnt/β-catenin signalling by the loss of TET1 is non-catalytic. Nonetheless, we observed that hyperactivated Nodal/Smad signalling can eventually activate Wnt/β-catenin through a signalling cross-talk (85). As further evidence of delayed Wnt activity induced by the loss of TET1 catalytic activity, Wnt pathway inhibition using XAV in *Tet1* MUT cells can completely repress expression of mesoderm and endoderm fate genes, and abolish ectopic H3K27me3 deposition, suggesting that 5mC oxidation by TET1 sustains the repression of Wnt signalling during late gastrulation. Though not as potent as in *Tet1* KO cells, the ‘leaky’ Nodal signals activating Wnt/β-catenin are sufficient to convert a sub-population to adopt mesoderm and endoderm fate in *Tet1* MUT.

In our previous ATAC-seq analysis for TET1-dependent DARs during naive (ESC) to formative (EpiLC) pluripotency transition, we detected mostly loss-in-KO regions in EpiLCs that were TET1 bound sites that gained accessibility in WT EpiLCs but failed to do so in *Tet1* KO, of which only a subset were associated with neural lineage enhancers (16). The minority of gain-in-KO sites were predominantly indirect targets that were already differentially accessible in naive ESCs. In this study, we examined cells further along their lineage bifurcation trajectory and found roughly equal numbers of close-in-KO and open-in-KO sites appearing by day 3, which are distal regulatory regions harbouring respectively ectoderm-specific and primitive streak-specific TF motifs. This dichotomy is strengthened by the preferential occupancy of TET1 to the ectoderm enhancers in the close-in-KO regions and absence of TET1 binding to open-in-KO regions. Furthermore, the chromatin state of these regions can be modulated both by inhibiting or activating Wnt signalling. These results suggest that TET1 sustains the accessibility of neuroectoderm enhancers during lineage priming and gastrulation onset. Consistent with a ‘neural default’ model, a recent single-cell multi-omics analysis during mouse gastrulation showed that the methylation and accessibility landscapes of ectodermal cells are already established in the early epiblast (9,86). Our temporal analysis suggests that loss of chromatin accessibility at ‘neural default’ enhancers then allows opening of alternative enhancers that promote primitive streak fates in response to signalling cues, suggesting that the ingression of epiblast cells into the primitive streak requires fundamentally the loss of full length TET1 expression. The precise non-catalytic mechanism by which TET1 maintains accessible chromatin at distal regulatory elements remains to be elucidated. Previous studies suggest the involvement of neural fate-specific TFs such as ZIC2 and EGR1 that recruit TET1 to enhancer sites (16,87), where TET1 may function as a binding partner of other chromatin regulators such as the O-linked N-acetylglucosamine (O-GlcNAc) transferase (88–90), or regulate promoter-enhancer chromatin looping.

It has been postulated that enhancer demethylation by TET enzymes and subsequently enhancer activation drives cellular fate in gastrulation (9). However, a causal link between enhancer demethylation and gene expression has been difficult to prove experimentally. In this study, selective loss of TET1 catalytic activity in *Tet1* MUT cells resulted in DNA hypermethylation as early as day 2 of differentiation at distal close-in-KO regions without inducing cell fate-altering chromatin accessibility changes at day 3. Presumably, the presence of catalytic dead TET1 at neural fate enhancers is compatible with *de novo* DNA methylation (80,91). The uncoupling of DNA methylation and chromatin accessibility in *Tet1* MUT cells suggests that 5mC oxidation at enhancers may only be a passive by-product of the dominant non-catalytic regulatory function of TET1. In agreement, a study that simultaneously profiled chromatin accessibility and DNA methylation from single DNA fragments in a monocyte-to- macrophage differentiation model, observed prolonged DNA methylation states at nascent open chromatin regions associated with transcriptional activation, suggesting that an uncoupling of DNA methylation from chromatin accessibility and gene activity may be a general mechanism (92). However, our analysis at a later time-point (day 5, post-gastrulation) revealed a class of *de novo* accessible enhancer loci, where DNA hypermethylation in KO was coupled with reduced chromatin opening, in line with an inverse correlation between DNA methylation and chromatin accessibility. To reconcile these findings, we propose that the persistence of DNA methylation at distal enhancers at lineage priming is not sufficient to induce chromatin closure of loci that were previously opened, whereas the loss of DNA methylation is more tightly linked with chromatin opening of *de novo* accessible enhancers at post-gastrulation. Like in its interaction with PRC2, TET1 may again be switching from a non-catalytic to a catalytic regulation of chromatin accessibility at distal enhancers during peri-gastrulation. Thus, the functional impact of DNA methylation changes on gene expression depends on CpG densities, which are generally much lower at distal enhancers than at proximal promoters, and also on the cellular context and developmental stage.

The signatures of DNA hypermethylation induced by TET1 dysfunction, when appearing latent in early progenitor cells, may have an impact on cellular functions later in development. We observed that *de novo* DNA hypermethylated regions increased in numbers as cells differentiated *in vitro* from day 2 to day 5 upon exogenous Wnt inhibition, largely due to DNA hypomethylation at intergenic loci in WT cells but persistence of methylation in KO post-gastrulation. Because there is lack of detectable TET activity at the later time-points, these results suggest that the engagement of TET1 in WT ESCs, particularly at neuroectodermal enhancers, marks a class of genomic loci for a subsequent loss of DNA methylation during differentiation. Perhaps, low basal levels of TET1 or TET3 during gastrulation may suffice to mediate DNA demethylation at these DMRs, or the early presence of TET1 may occlude DNMT and facilitate passive DNA demethylation during subsequent cell divisions. Moreover, the hypermethylation in KO observed at distal enhancer regions regulating fetal brain-specific genes at day 5 is associated with reduced chromatin accessibility, suggesting that the epigenetic defects may affect subsequent brain development. Supporting the notion of an early developmental origin of disease, we showed that promoter hyper methylation of brain-specific genes associated with postnatal neurological dysfunction in *Tet1*-deficient mice already exhibited gains in methylation in day 5 *Tet1* KO antNPCs, and in E8.5 anterior neural tissues and E11.5 brains (18,71,72). Therefore, previous phenotypes associated with TET1 dysfunction in adult mice may have an aetiology occurring as early as during peri-gastrulation stages.

**Figure 8.**
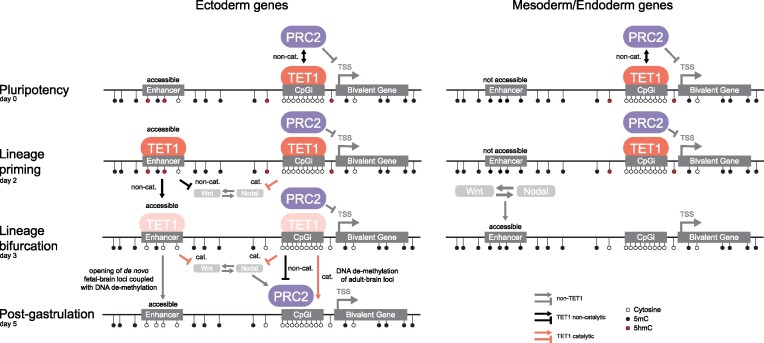
Catalytic and non-catalytic functions of TET1 separated by genomic context, lineage, and developmental stage. Unmethylated cytosine in white, 5-methyl-cytosine (5mC) in black, 5-hydroxy-methyl-cytosine (5hmC) in red. non-TET1 functions in grey lines, TET1 non-catalytic functions in black lines, TET1 non-catalytic functions in red lines.

Overall, our study resolves the seemingly paradoxical functions of TET1 in cell fate decision at early germ layer lineage bifurcation. We could partition the relative contributions of TET1’s catalytic and non-catalytic activities to distinct CpGi-associated and distal genomic contexts, where each mode of activity exerts different functional impacts and dominance depending on lineage and developmental stage (Figure 8). These results present a more nuanced view of the role of TET1 as a chromatin regulator in embryonic development. Rather than strictly acting as a ‘turn on’ switch at regulatory regions to activate gene expression, the catalytic and non-catalytic dual modes confer TET1 with a versatility to switch dynamically between ‘activator’ and ‘repressor’ roles at different genomic context and stages of development.

## DATA AVAILABILITY

All RNA-seq, 10xGenomics scRNA-seq, WGBS, ATAC-seq and CUT&RUN data are available at https://www.ncbi.nlm.nih.gov/geo/ with accession number GSE214845.

## Supplementary Material

gkad231_Supplemental_Files
