# Supplementary material for: Dual functions of TET1 in germ layer lineage bifurcation distinguished by genomic context and dependence on 5-methylcytosine oxidation
Source: Nucleic Acids Res. 2023 Apr 6;51(11):5469–98. doi: 10.1093/nar/gkad231 (PMC10287924; doi:10.1093/nar/gkad231)
Supplement: gkad231_Supplemental_Files [file gkad231_supplemental_files.zip › supplementary_figure1-7_proof.pdf]

van der Veer et al., Figure S1

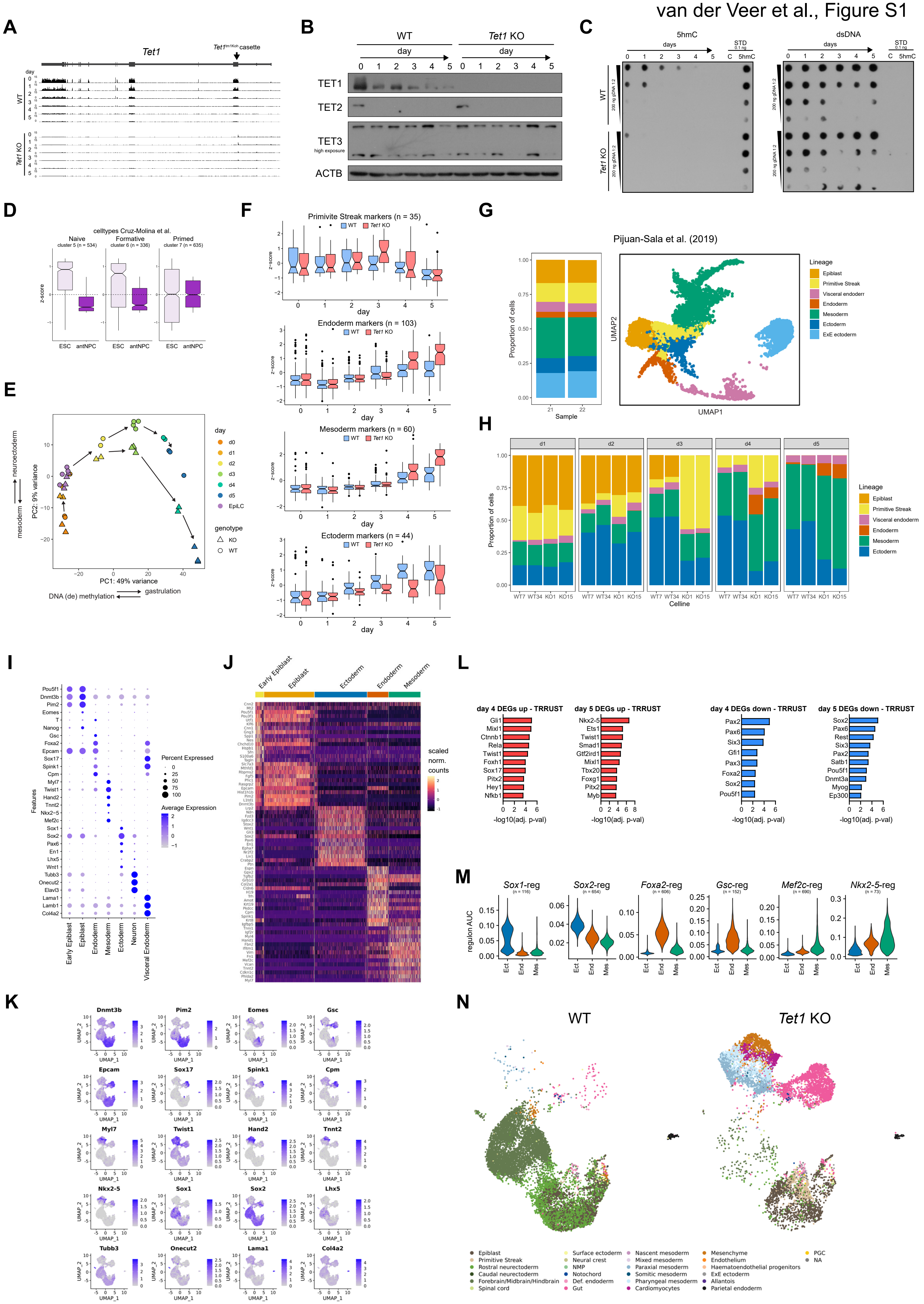

Figure S1.

A) Integrated genomics viewer (IGV) tracks of RNA-seq normalized reads at the *Tet1* locus in representative *Tet1* KO and WT samples during the differentiation time-course. The start location of the *Tet1*^{tm1Koh} allele insertion is indicated. **B)** Western blot of TET1, TET2, and TET3 over time during neurobasal differentiation of *Tet1* KO and WT cells. Note, in order to show a band, the blot for TET3 required a high exposure. **C)** Example of 5hmC and double-stranded DNA dot blots. **D)** Z-score based on TPM of cluster genes in Figure 1F in ESC or antNPC bulk RNA-seq datasets (26). **E)** PCA plot of bulk RNA-seq data obtained during the differentiation time course, incorporating a RNA-seq dataset from EpiLCs. **F)** Z-score based on TPM of lineage marker genes determined using mouse gastrulation scRNA-seq reference dataset (2). **G)** Lineage proportion and UMAP of mixed samples from the mouse gastrulation reference dataset used for deconvolution. **H)** Bulk time-course RNA-seq data deconvoluted to estimate proportion of cell types per cell line per day, using the mouse gastrulation reference dataset. **I)** Expression level of lineage markers per UMAP cluster defined in Figure 1J using the integrated scRNA-seq dataset of *Tet1* KO vs WT cells from day 3 and day 5. **J)** Heatmap of unsupervised markers per UMAP cluster. The small Neuron and Visceral Endoderm clusters are left out for clarity. **K)** Expression levels of additional lineage markers projected on UMAP space and expressed as scaled normalized counts. **L)** Top 10 transcription factors (TFs) predicted to regulate *Tet1* KO vs WT DEGs at day 4 and day 5 using TRRUST. **M)** Regulon activity for lineage markers per differentiated cluster expressed as regulon area under the curve (AUC). Regulon size (number of genes in each regulon) is indicated within parentheses. **N)** UMAP with cell types annotated using the mouse gastrulation reference. For clarity the UMAP is split between *Tet1* KO and WT cells.

van der Veer et al., Figure S2

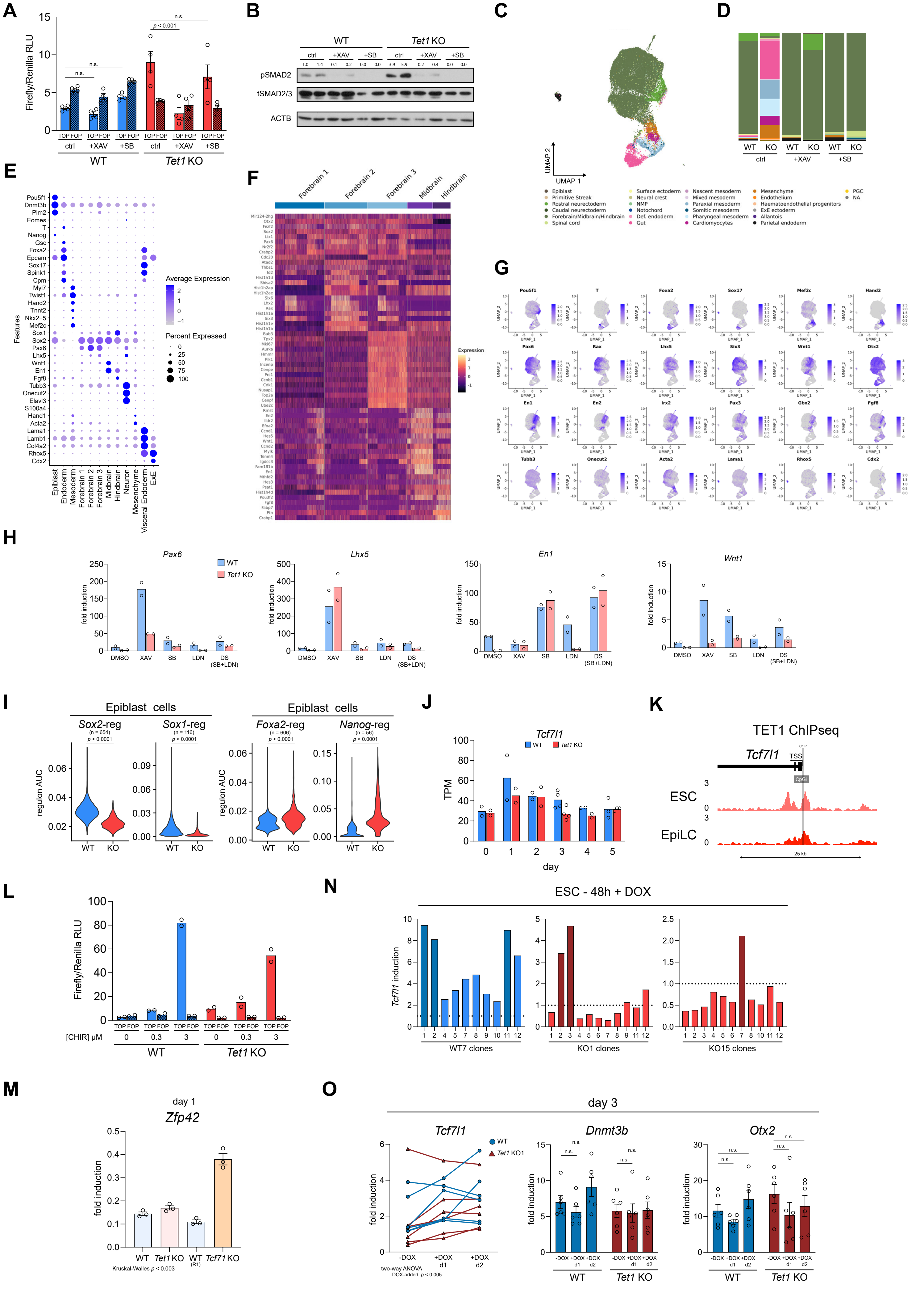

Figure S2.

A) Wnt activity assay using a TOP/FOP-flash reporter at day 3 of differentiation in *Tet1* KO and WT cells treated with 5 µM of the Wnt inhibitor XAV939 (XAV), 2.5 µM of the Nodal signalling inhibitor SB431542 (SB), or similar volume of DMSO as vehicle control from day 2. Data are shown as mean ± SEM of n = 4 biological replicates, using two different ESC lines per genotype. **B)** pSMAD2 western blot as readout for Nodal signalling in cells at day 3 of differentiation upon inhibitor treatment as in A. Bands are quantified by using ImageJ and the values normalized to total SMAD2 (top band of tSMAD2/3) are indicated above the blot. **C-D)** UMAP **(C)** and cell type proportion **(D)** of day 5 cells annotated using the mouse gastrulation reference atlas. **E)** Expression levels of lineage markers per UMAP cluster defined using day 5 scRNA-seq datasets shown in Figure 2E-G. **F)** Heatmap of unsupervised markers per cluster. Only forebrain, midbrain and hindbrain clusters are shown for clarity. **G)** Expression levels of lineage markers projected on UMAP space and expressed as scaled normalized counts. **H)** Gene expression based on qPCR analysis of ectoderm markers (*Pax6*, *Lhx5*, *En1*, and *Wnt1*) in *Tet1* KO and WT cells at day 5 following treatment with 5 µM XAV, 2.5 µM SB, 0.1 µM of the BMP signalling inhibitor LDN193189 (LDN), and a combination of 2.5 µM SB + 0.1 µM of LDN, or DMSO as vehicle control starting from day 2. Data are shown as the mean of two different ESC lines per genotype. **I)** Activity of regulons that are differentially activated in *Tet1* KO vs WT Epiblast cluster cells. **J)** Expression of *Tcf7l1* in both *Tet1* KO and WT cells during differentiation (without inhibitor treatment) from RNA-seq data. Data are shown as the mean of 4 samples at day 3, 3 samples at day 5 and otherwise 2 samples at all other time-points. **K)** IGV tracks of TET1 ChIP-seq data in naive ESCs and primed EpiLCs (15). **L)** Wnt activity assay using TOP/FOP-flash reporter at day 3 of differentiation in *Tet1* KO and WT cells treated for 24h with 0.3 µM or 3 µM Wnt activator CHIR99021 (CHIR) or DMSO as vehicle control. **M)** Expression of naive pluripotency marker *Zfp42* at day 1 of differentiation in *Tet1* KO, WT, *Tcf7l1* KO and R1 ESCs (same background as *Tcf7l1* KO cells) measured using qPCR. Data are shown as mean ± SEM of n = 3 independent differentiations using two ESC lines for *Tet1* KO and WT, and one ESC line for *Tcf7l1* KO and R1 control. **N)** Induction level of *Tcf7l1* in individual clonal ESC lines, treated with 2 µg/ml of DOX for 48h. Reference level (dotted line) is the average of clonal lines without DOX treatment. **O)** Expression of *Tcf7l1*, and primed pluripotency markers *Dnmt3b* and *Otx2*, at day 3 of differentiation in *Tet1* KO and WT ESCs over-expressing *Tcf7l1* after addition of 2 µg/ml of DOX at day 1 or day 2. Data are shown as mean ± SEM of n = 6 from 2 independent differentiations using 3 different ESC lines per genotype.

van der Veer et al., Figure S3

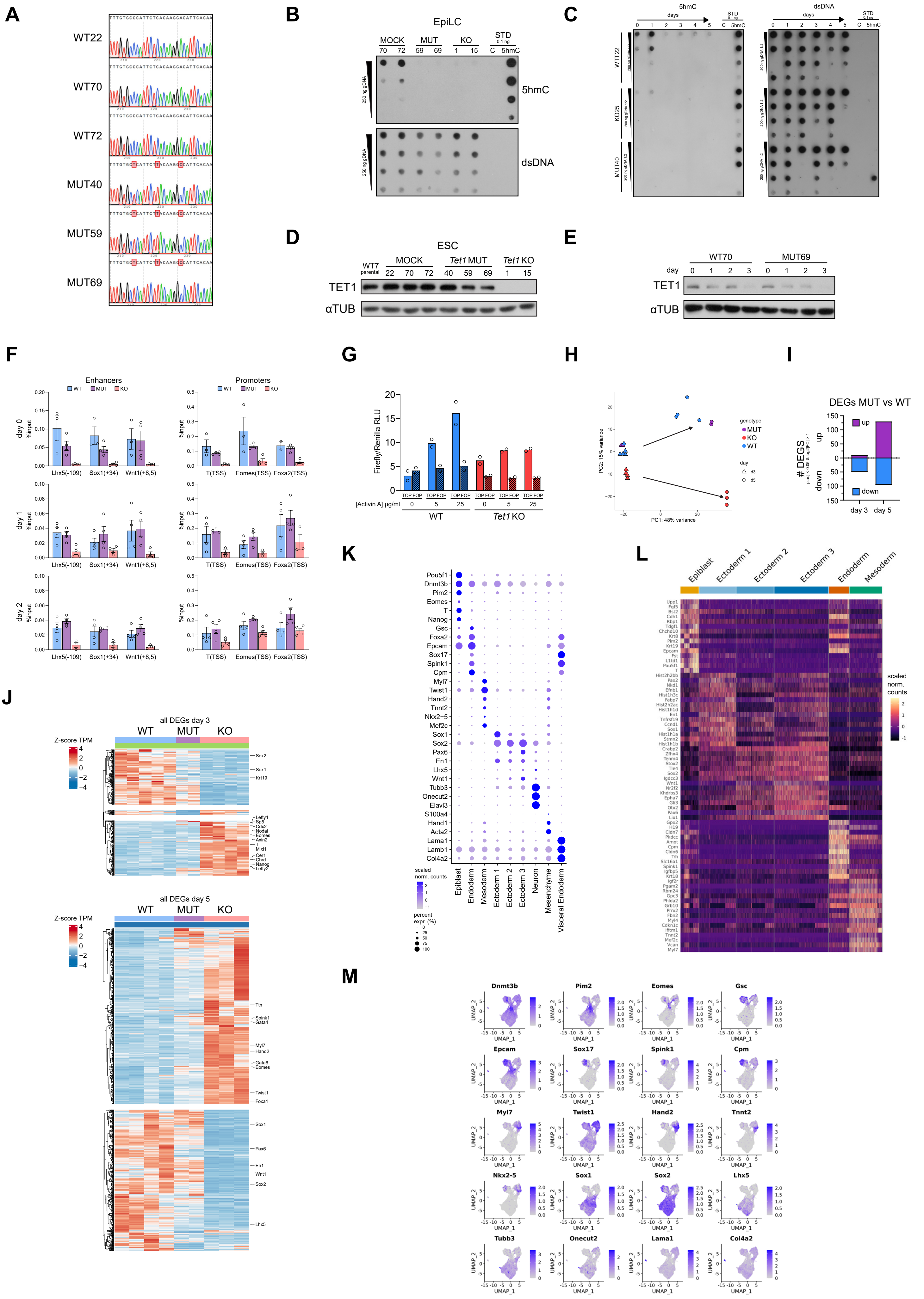

Figure S3.

A) Sanger sequencing of the locus targeted by homology directed repair in the three validated *Tet1* MUT and mock transfected (used as WT in this figure) clonal ESC lines. **B-C)** 5hmC and dsDNA dot blot analysis of WT (mock), *Tet1* mutant and *Tet1* KO ESCs converted *in vitro* into EpiLCs **(B)** or antNPC over the differentiation time-course **(C)**. Numbers above each lane in B denote clone IDs. **D-E)** Western blot for TET1 in ESCs in serum plus LIF cultures **(D)** or at day 0 (ESC), 1, 2, 3 of differentiation **(E).** WT7 is the parental WT ESC line. **F)** ChIP-qPCR analysis of TET1 binding at selected ectoderm enhancers (*Lhx5*, *Sox1*, *Wnt1*; distance in kb from TSS indicated within parentheses) or primitive streak gene promoters (*T*, *Eomes*, *Foxa2*) at day 0 (ESC), 1, and 2 of differentiation. *Tet1* KO lines serve as negative controls. Data are shown as mean ± SEM of n = 3 (WT) or 4 (MUT and KO) biological replicates using 3 ESC lines per genotype in two independent differentiations. TSS, transcription start site. **G)** Wnt activity assay using TOP/FOP-Flash reporter at day 3 of antNPC differentiation in *Tet1* KO and WT cells after a 24h treatment with 5 ng/ml or 25 ng/ml of Nodal signalling agonist Activin A. Data are shown as the mean of two experiments using independent ESC lines per genotype. **H)** PCA plot of bulk RNA-seq samples collected at day 3 and day 5 of neurobasal differentiation of *Tet1* KO, MUT, and WT samples. **I)** Number of DEGs between *Tet1* MUT vs WT at day 3 and day 5. **J)** Heatmap of all unique DEGs (MUT vs WT or KO vs WT only) at day 3 and day 5 expressed as Z-score of RNA-seq TPM. **K)** Expression level of lineage markers per UMAP cluster based on day 5 scRNA-seq datasets including *Tet1* KO, MUT, mock and parental WT cells. **L)** Heatmap of unsupervised markers per cluster. Visceral endoderm and neuron clusters are left out for clarity. **M)** Expression levels of additional lineage markers projected on UMAP space and expressed as scaled normalized counts.

van der Veer et al., Figure S4

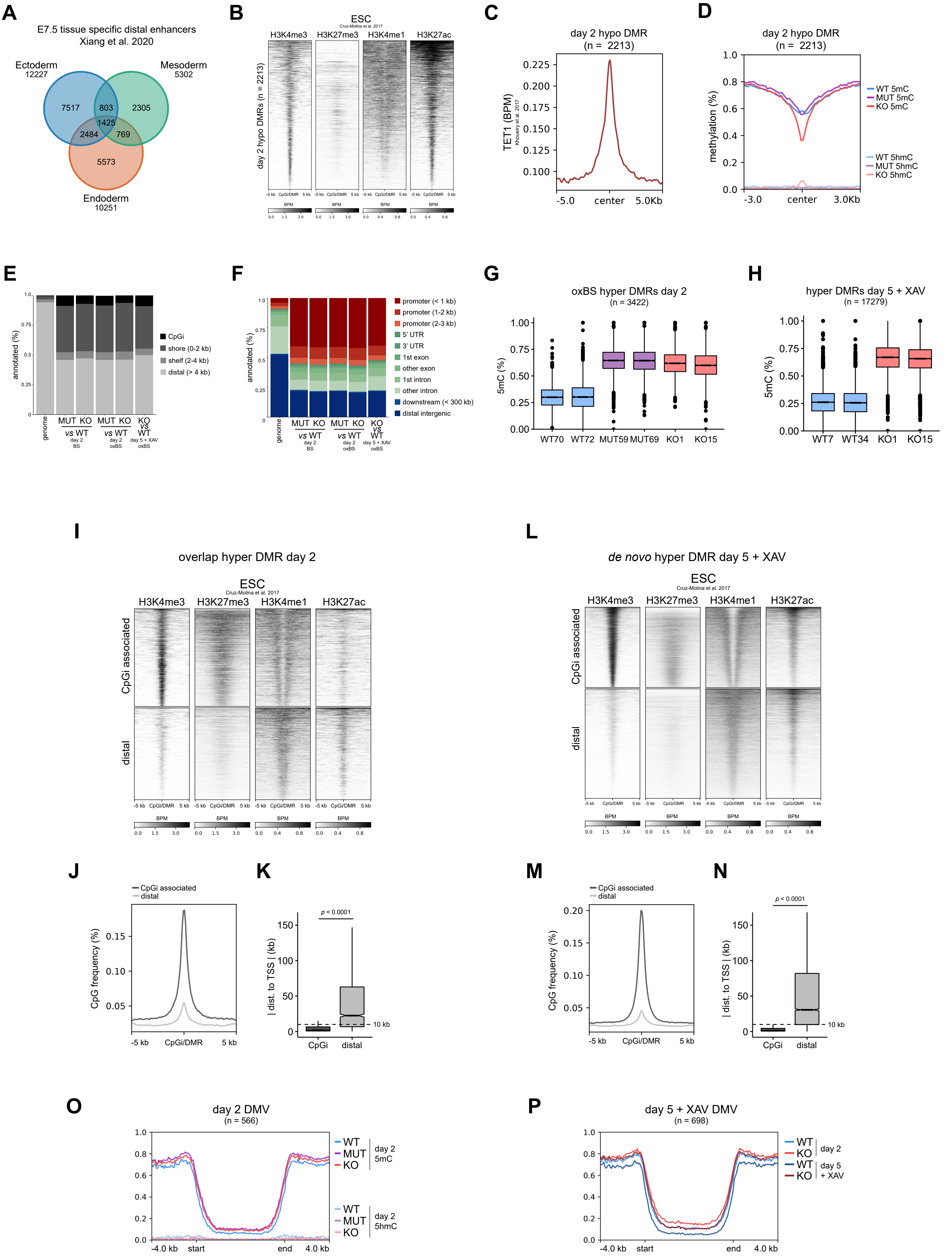

Figure S4.

A) Venn diagram of distal H3K27ac peaks called per E7.5 germ layer tissue. Tissue-specific enhancers are considered as peaks that are not present in any of the other E7.5 tissues (45). **B)** Heatmaps of H3K4me3, H3K27me3, H3K4me1, H3K27ac ChIP-seq histone marks in ESCs at hypo DMRs in *Tet1* KO cells (26). **C)** Profile plot of TET1 binding at day 2 hypo DMRs in EpiLCs (15). **D)** 5mC and 5hmC levels inferred from the combined analysis of BS and oxBS datasets at day 2 hypo DMRs in *Tet1* KO, MUT, and WT cells. **E-F)** Genomic features based on CpGi proximity **(E)** and gene structure **(F)** annotation of DMRs between *Tet1* KO vs WT, or MUT vs WT, determined from BS and oxBS datasets, compared to background of the entire genome. **G)** Boxplot of average 5mC rate at hyper DMRs per cell line at day 2 of differentiation. **H)** Boxplot of average 5mC methylation levels at day 5 (+XAV) hyper DMRs per cell line. **I)** Heatmaps of H3K4me3, H3K27me3, H3K4me1, H3K27ac ChIP-seq in ESCs (26) at TET1-dependent day 2 hyper DMRs **J)** Profile plot for CpG frequencies at day 2 hyper DMRs. **K)** Distance to nearest TSS for CpGi-associated and distal day 2 hyper DMRs. **L)** Heatmaps of H3K4me3, H3K27me3, H3K4me1, H3K27ac ChIP-seq in ESCs (26) at TET1-dependent day 5 (+XAV) hyper DMRs. **M)** Profile plot for CpG frequencies at day 5 (+XAV) hyper DMRs. In I, J, L, and M, CpGi-associated DMRs are centred at the nearest associated CpGi, while intergenic DMRs are centred at the DMR itself. **N)** Distance to nearest TSS for CpGi-associated and distal day 5 (+XAV) hyper DMRs. **O-P)** Profile plots of 5mC and 5hmC over DNA methylation valleys (DMVs) in WT, MUT, and KO cells at day 2 **(O)** and day 5 (+XAV) **(P)**.

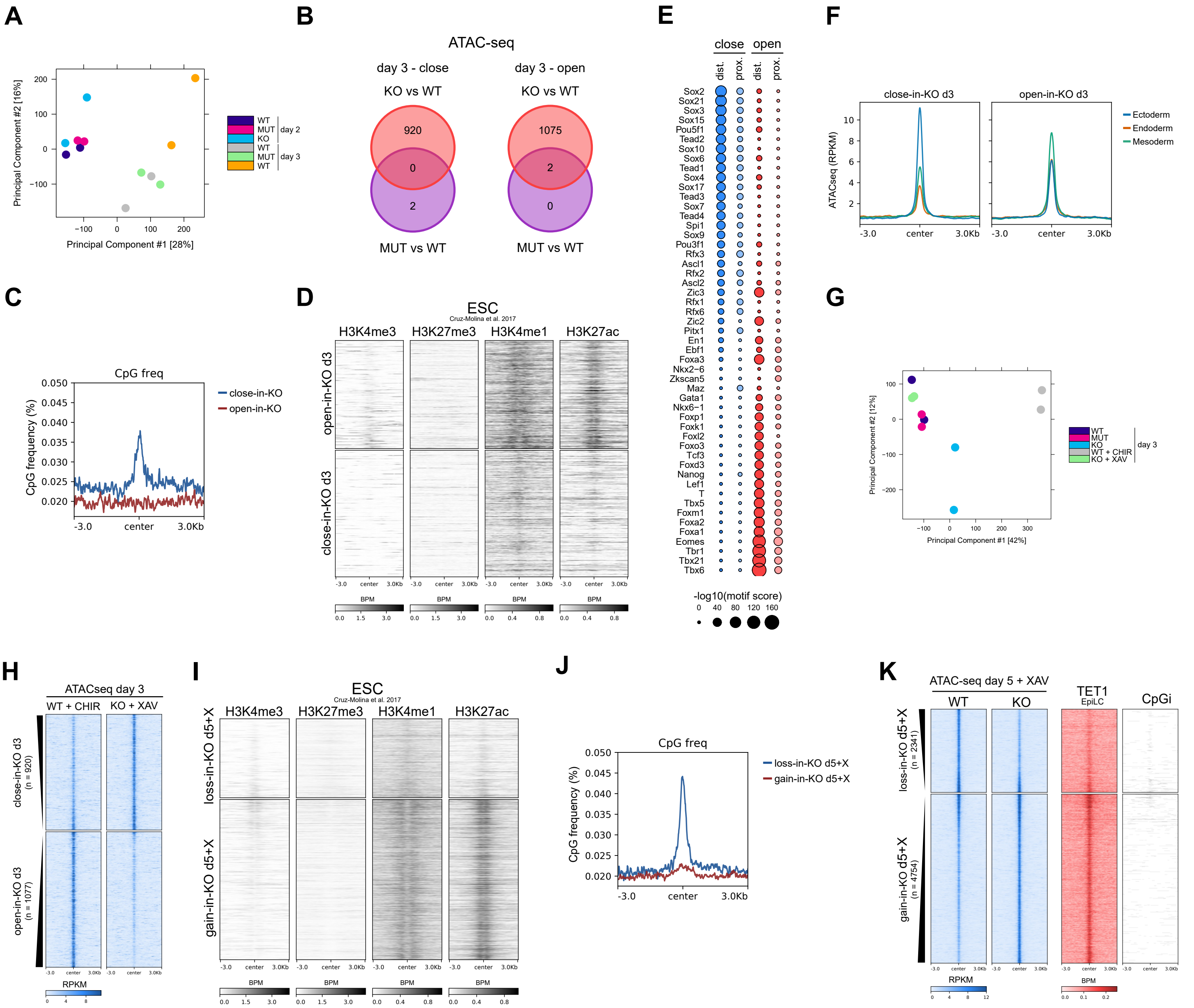
A
Principal Component #2 [16%]
Principal Component #1 [28%]
WT
MUT
KO
WT
MUT
WT
day 2
day 3
B
ATAC-seq
day 3 - close
day 3 - open
KO vs WT
KO vs WT
920
0
2
1075
2
0
MUT vs WT
MUT vs WT
C
CpG freq
close-in-KO
open-in-KO
CpG frequency (%)
-3.0
center
3.0Kb
D
ESC
Cruz-Molina et al. 2017
H3K4me3
H3K27me3
H3K4me1
H3K27ac
open-in-KO d3
close-in-KO d3
BPM
E
close
open
dist.
prox.
dist.
prox.
Sox2
Sox21
Sox3
Sox15
Pou5f1
Tead2
Sox10
Sox6
Tead1
Sox4
Sox17
Tead3
Sox7
Tead4
Spi1
Sox9
Pou3f1
Rfx3
Ascl1
Rfx2
Ascl2
Zic3
Rfx1
Rfx6
Zic2
Pitx1
En1
Ebf1
Foxa3
Nkx2−6
Zkscan5
Maz
Gata1
Nkx6−1
Foxp1
Foxk1
Foxl2
Foxo3
Tcf3
Foxd3
Nanog
Lef1
T
Tbx5
Foxm1
Foxa2
Foxa1
Eomes
Tbr1
Tbx21
Tbx6
-log10(motif score)
0 40 80 120 160
F
close-in-KO d3
open-in-KO d3
ATACseq (RPKM)
Ectoderm
Endoderm
Mesoderm
G
Principal Component #2 [12%]
Principal Component #1 [42%]
WT
MUT
KO
WT + CHIR
KO + XAV
day 3
H
ATACseq day 3
WT + CHIR
KO + XAV
close-in-KO d3
(n = 920)
open-in-KO d3
(n = 1077)
RPKM
I
ESC
Cruz-Molina et al. 2017
H3K4me3
H3K27me3
H3K4me1
H3K27ac
loss-in-KO d5+X
gain-in-KO d5+X
BPM
J
CpG freq
loss-in-KO d5+X
gain-in-KO d5+X
CpG frequency (%)
K
ATAC-seq day 5 + XAV
WT
KO
TET1
EpiLC
CpGi
loss-in-KO d5+X
(n = 2341)
gain-in-KO d5+X
(n = 4754)
RPKM
BPM

Figure S5.

A) PCA plot of *Tet1* KO, MUT and WT ATAC-seq samples at day 2 and day 3 of differentiation. **B)** Venn diagram of overlap between close-in-KO and close-in-MUT (left panel), and between open-in-KO and open-in-MUT (right panel) differentially accessible regions (DARs). **C)** Profiles of CpG frequencies at close-in-KO or open-in-KO DARs at day 3. **D)** Heatmaps of H3K4me3, H3K27me3, H3K4me1, H3K27ac ChIPseq in ESCs (26) at close-in-KO or open-in-KO day 3 DARs. **E)** Motif enrichment score for top 20 distal and proximal motifs in close-in-KO and open-in-KO at day 3. **F)** Profile plots of ATAC-seq signals in RPKM in E7.5 germ layer-specific tissues centred at close-in-KO and open-in-KO day 3 DARs. **G)** PCA plot of *Tet1* KO, MUT, WT, WT cells treated for 24h with 3 µM of the Wnt activator CHIR99021 (CHIR), and KO cells treated with the Wnt inhibitor XAV939 (XAV) at day 3 of differentiation. **H)** Heatmap of ATAC-seq signals in RPKM at day 3 in WT + CHIR or KO + XAV, centred at day 3 close-in-KO (top panels) and open-in-KO (bottom panels) DARs and ordered by fold change of differential accessibility. **I)** Heatmaps of histone marks H3K4me3, H3K27me3, H3K4me1, H3K27ac ChIP-seq in ESCs (26) at close-in-KO or open-in-KO DARs at day 5 (+XAV). **J)** Profile plot of CpG frequencies at close-in-KO and open-in-KO day 5 (+XAV) DARs. **K)** Heatmap of ATAC-seq signals in RPKM in KO and WT cells at day 5 (+XAV), TET1 binding in BPM in EpiLCs, and CpGi frequency centred at day 5 (+XAV) close-in-KO (top panels) and open-in-KO (bottom panels). DARs ordered by fold change of differential accessibility.

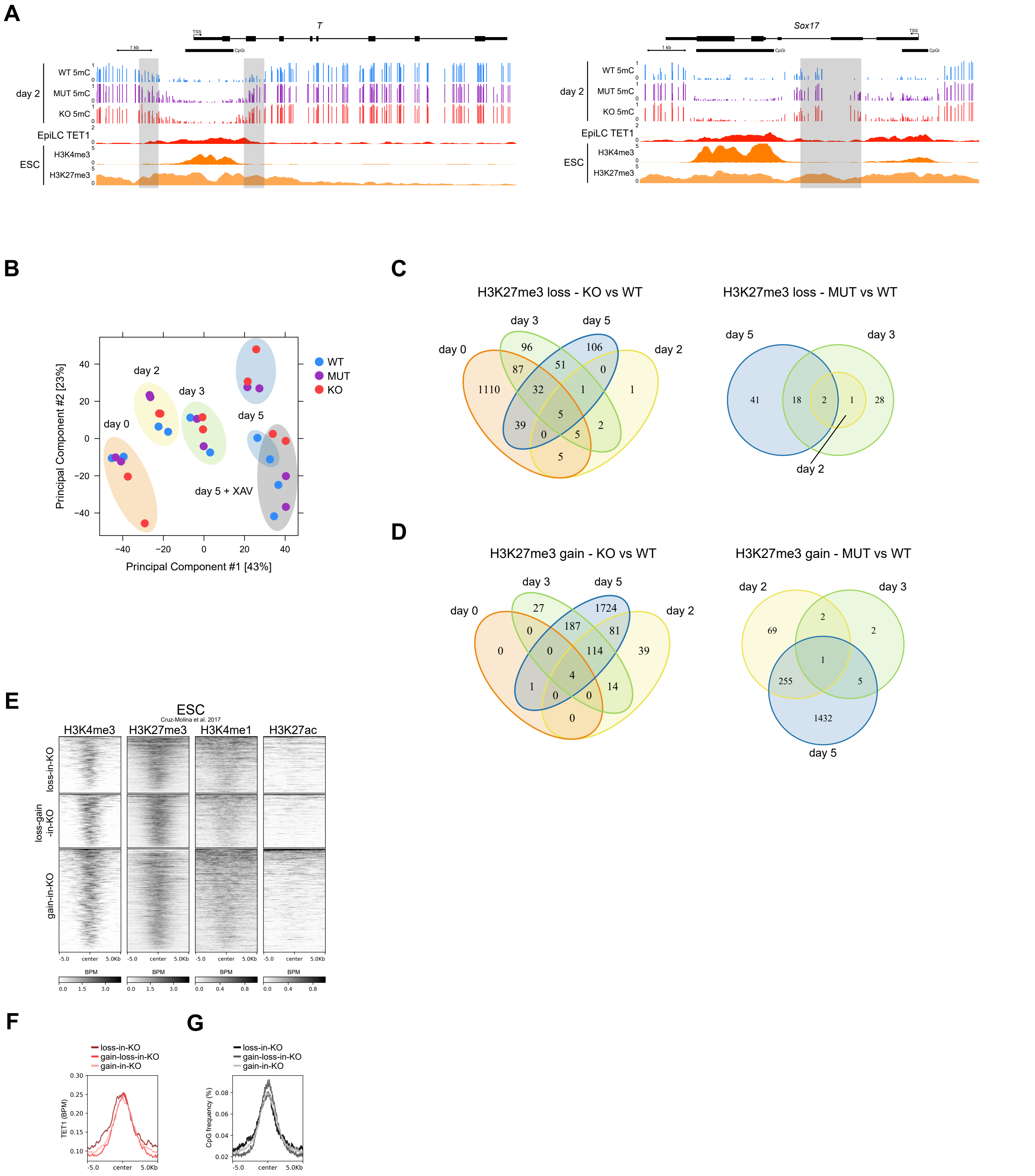
A
T
TSS
1 kb
CpGi
day 2
WT 5mC
MUT 5mC
KO 5mC
EpiLC TET1
ESC
H3K4me3
H3K27me3
Sox17
B
Principal Component #2 [23%]
Principal Component #1 [43%]
day 0
day 2
day 3
day 5
day 5 + XAV
WT
MUT
KO
C
H3K27me3 loss - KO vs WT
day 0
day 3
day 5
day 2
1110
96
106
1
87
51
0
32
1
39
5
2
0
5
5
H3K27me3 loss - MUT vs WT
day 5
day 3
day 2
41
18
2
1
28
D
H3K27me3 gain - KO vs WT
day 0
day 3
day 5
day 2
0
27
1724
39
0
187
81
0
114
4
1
14
0
0
0
H3K27me3 gain - MUT vs WT
day 2
day 3
day 5
69
2
2
1
255
5
1432
E
ESC
Cruz-Molina et al. 2017
H3K4me3
H3K27me3
H3K4me1
H3K27ac
loss-in-KO
loss-gain -in-KO
gain-in-KO
-5.0
center
5.0Kb
BPM
F
loss-in-KO
gain-loss-in-KO
gain-in-KO
TET1 (BPM)
G
loss-in-KO
gain-loss-in-KO
gain-in-KO
CpG frequency (%)

Figure S6.

A) IGV tracks of 5mC methylation levels in day 2 cells, TET1 binding in EpiLCs, and H3K4me3 and H3K27me3 ChIP-seq bivalent histone marks in ESCs at the example loci *T* and *Sox17*. DMRs are indicated with black. **B)** PCA plot of H3K27me3 CUT&RUN at day 0, day 2, day 3, day 5, day 5 (+XAV) in *Tet1* KO, MUT, and WT. **C-D)** Venn diagram of differential H3K27me3 enrichment regions. **E)** Heatmaps of H3K4me3, H3K27me3, H3K4me1, H3K27ac ChIP-seq in ESCs (26) at regions with significant H3K27me3 loss-in-KO, gain-loss-in-KO, gain-in-KO. **F-G)** TET1 binding in EpiLCs **(F)** or CpG frequency **(G)** at loss-in-KO, gain-loss-in-KO, and gain-in-KO regions.

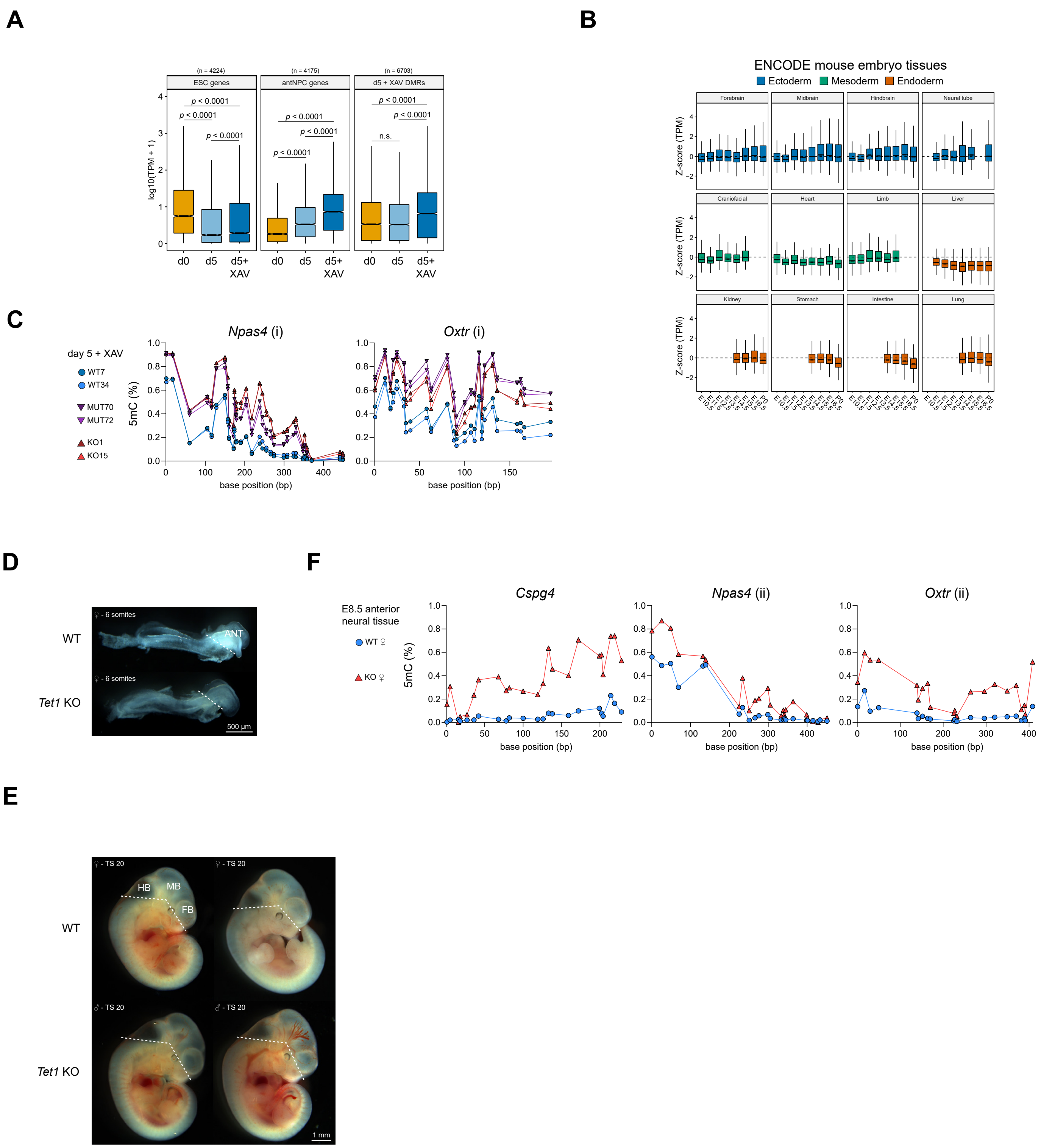
A
B
C
D
E
F
(n = 4224)
(n = 4175)
(n = 6703)
ESC genes
antNPC genes
d5 + XAV DMRs
log10(TPM + 1)
p < 0.0001
n.s.
d0
d5
d5+ XAV
ENCODE mouse embryo tissues
Ectoderm
Mesoderm
Endoderm
Forebrain
Midbrain
Hindbrain
Neural tube
Craniofacial
Heart
Limb
Liver
Kidney
Stomach
Intestine
Lung
Z-score (TPM)
day 5 + XAV
WT7
WT34
MUT70
MUT72
KO1
KO15
Npas4 (i)
Oxtr (i)
5mC (%)
base position (bp)
WT
Tet1 KO
♀ - 6 somites
ANT
500 µm
E8.5 anterior neural tissue
WT ♀
KO ♀
Cspg4
Npas4 (ii)
Oxtr (ii)
♀ - TS 20
♂ - TS 20
HB
MB
FB
1 mm

Figure S7.

A) Boxplots of log_{10}(TPM) of ESC-specific genes, antNPC-specific genes, and genes associated with d5 (+XAV) hyper DMRs **B)** Box-plots of ENCODE RNA-seq TPM Z-scores of genes that are within 10 kb of day 5 (+XAV) hyper DMRs per embryonic tissues ranging from E10.5 until P0 (30). The different colours indicate the germ layer lineage origin of each tissue (ectoderm, mesoderm, or endoderm). **C)** 5mC methylation levels at region (i) of *Npas4* and *Oxtr*, in *Tet1* KO, MUT, and WT day 5 (+XAV) antNPCs measured using amplicon bisulfite sequencing. **D)** *Tet1* KO and WT E8.5 embryos used to collect headfolds. Dotted line indicates dissection plane, ANT = anterior neural (headfold) tissue, scalebar = 500 µm **E)** E11.5 *Tet1* KO and WT embryos used to collect embryonic brain tissues. Dotted line indicates dissection plane, HB = hindbrain, MB = midbrain, FB = forebrain, scalebar = 1 mm. **F)** 5mC methylation levels at promoters of *Cspg4*, *Npas4* (region ii), and *Oxtr* (region ii) in *Tet1* KO and WT E8.5 anterior neural (headfold) tissues from sex and stage-matched littermates, measured using amplicon bisulfite sequencing.
